# New Insights into lncRNAs in Aβ Cascade Hypothesis of Alzheimer’s Disease

**DOI:** 10.3390/biom12121802

**Published:** 2022-12-02

**Authors:** Yitong Hao, Bo Xie, Xiaoshu Fu, Rong Xu, Yu Yang

**Affiliations:** 1 Department of Neurology and Neuroscience Center, The First Hospital of Jilin University, Changchun 130021, China; 2 Department of Hepatobiliary and Pancreatic Surgery, The First Hospital of Jilin University, Changchun 130021, China

**Keywords:** Alzheimer’s disease, Aβ, lncRNAs, biomarkers, therapeutics

## Abstract

Alzheimer’s disease (AD) is the most common type of dementia, but its pathogenesis is not fully understood, and effective drugs to treat or reverse the progression of the disease are lacking. Long noncoding RNAs (lncRNAs) are abnormally expressed and deregulated in AD and are closely related to the occurrence and development of AD. In addition, the high tissue specificity and spatiotemporal specificity make lncRNAs particularly attractive as diagnostic biomarkers and specific therapeutic targets. Therefore, an in-depth understanding of the regulatory mechanisms of lncRNAs in AD is essential for developing new treatment strategies. In this review, we discuss the unique regulatory functions of lncRNAs in AD, ranging from Aβ production to clearance, with a focus on their interaction with critical molecules. Additionally, we highlight the advantages and challenges of using lncRNAs as biomarkers for diagnosis or therapeutic targets in AD and present future perspectives in clinical practice.

## 1. Introduction

Alzheimer’s disease (AD) is the most common type of dementia. The main pathophysiological features of AD are β-amyloid peptide (Aβ) aggregation forming senile plaques (SPs) and phosphorylated tau (p-tau) aggregation leading to neurofibrillary tangles (NFTs) [1]. The amyloid cascade hypothesis is thought to play an extremely important role in the pathogenesis of AD [2]. Aβ, as the initiating link in AD pathogenesis, can trigger a series of downstream events, such as tau protein hyperphosphorylation, oxidative stress, inflammatory response, and synaptic dysfunction [3,4,5]. Aβ oligomer theory suggests that soluble Aβ oligomers (oAβ), a major source of neurotoxicity, can cause neuronal damage at the nanomolar level [6]. Hence, Aβ has been one of the most popular targets in AD drug research. However, clinical drugs targeting Aβ have not yet produced the desired effect, and clinical diagnosis and drug treatment for Aβ are facing considerable challenges [2]. Therefore, an urgent need remains to explore further the causes of the imbalance in Aβ production and the clearance in AD patients, and to understand the molecular mechanisms underlying AD pathology better to develop new therapeutic targets.

Approximately 75% of the human genome is transcribed, but only 2% of the transcribed genes encode mRNAs with protein synthesis potential. According to the ENCODE program, the vast majority of transcripts produce lncRNAs, of which up to 40% are preferentially expressed in the brain [7,8]. Initially, lncRNAs were regarded as by-products of RNA polymerase II transcription, lacking biological function, so they were not widely appreciated [9,10]. However, sensitive and precise high-throughput gene transcriptome sequencing revealed that the expression levels of many lncRNAs are significantly altered in the brains of AD patients [11,12,13]. The expression levels of some lncRNAs in the brain, blood, or cerebrospinal fluid of AD patients correlate with the severity of the disease [13,14]. In vivo and in vitro experiments verified that lncRNAs play an important role in the Aβ cascade hypothesis, affecting the production, clearance, and neurotoxicity of Aβ. This suggests that lncRNAs play an integral role in the pathogenesis and staging of AD and may become new therapeutic targets and biomarkers for AD. Therefore, in this paper, we summarize the exact functions and related mechanisms of lncRNAs in Aβ deposition and neurotoxicity, aiming to provide a theoretical basis for lncRNAs to become diagnostic markers and therapeutic targets for AD.

## 2. lncRNAs

Although the human genome has been extensively transcribed, only about 2% of RNAs encode proteins [15,16]. Most expressed transcripts do not encode proteins, with transcripts of 200 nt in length usually defined as lncRNAs [17,18]. No unified standard exists for the classification of lncRNAs. According to the position of lncRNAs concerning protein-coding genes, they can be classified into five major groups [18,19,20,21]: (I) Sense lncRNA: transcribed from a protein-coding gene, transcribed in the same direction as the neighboring mRNA, and overlapping at least one protein-coding exon; (II) Antisense lncRNA: transcribed from the antisense strand of a protein-coding gene, transcribed in the opposite direction of the mRNA, often overlapping one or more protein-coding introns or exons; (III) Bidirectional lncRNA: transcription start site is closely related to the transcription start site of the protein-coding gene on the opposite strand, which is located within 1 kb of the promoter of the neighboring gene and can be transcribed from both the same and opposite directions as the neighboring mRNA; (IV) Intronic transcript lncRNA: transcribed from the intronic region of the protein-coding gene; (V) Intergenic lncRNA: transcribed from the region between two protein-coding genes, with its own promoter. Structurally, lncRNAs usually have a mC cap at the 5’ end, either with or without a poly-A tail at the 3’ end [22,23]. lncRNAs were initially regarded as unstable, but this only applies to a few lncRNAs [24], most of which are stabilized by polyadenylation [25,26]. Nonpolyadenylated lncRNAs can be stabilized by a triple-helix structure in their 3’ end [27]. These triple-helical structures at the 3’ end promote the effective nuclear export of lncRNAs and have a stabilizing effect [28]. lncRNAs are poorly conserved in sequence [29], with about 12% of lncRNAs found in organisms other than humans. Nevertheless, some lncRNAs exhibit high conservation and may play comparable roles across species [30]. Researchers recently discovered that, although some important lncRNAs show sequence or positional conservation between human and mouse embryonic stem cells, they are differently processed and thus localize to different subcellular compartments, ultimately performing different functions in mouse and human cells [31]. This study showed that lncRNA sequence conservation does not always translate into conserved functional roles and that different processing and binding molecules of lncRNAs can substantially affect their subcellular distribution and function. Many lncRNAs are found in more than one organelle or compartment [32,33], hindering assessments of lncRNA function. The regulatory mechanisms of lncRNAs are complex. As the research has progressed, evidence has suggested that lncRNAs may be involved in the epigenetic, transcription, and post-transcriptional regulation of the target gene. 

The human genome encodes tens of thousands of lncRNAs, most of which are specifically expressed in the brain [8]. The results of transcriptome analysis of postmortem human brains revealed that the levels of multiple lncRNAs are significantly altered in the brains of AD patients [11,13]. In combination with the results obtained from mouse models and cell lines [34], increasing numbers of the mechanisms of action of lncRNAs associated with AD pathogenesis have been elucidated, especially in Aβ metabolism, where lncRNAs can affect Aβ production and clearance through various regulatory mechanisms [23,35], implicating lncRNAs in playing an important role in the development and progression of AD. In addition, using real-time quantitative PCR(qRT-PCR), researchers have found that these functional lncRNAs are also dysregulated in the cerebrospinal fluid and blood of AD patients and correlate with the degree of cognitive impairment in AD [36,37]. This finding not only suggests that functional lncRNAs are further involved in the pathogenesis of AD but also highlights their potential as a therapeutic target and diagnostic biomarker for AD.

## 3. Regulatory Effects of lncRNAs on Aβ Production

APP is a type I membrane protein widely present in various tissues and is concentrated at the synaptic sites of neurons. Its active, larger N-terminal is located outside the cell, and its shorter C-terminal is located inside the cell, where the Aβ fragment is located in the transmembrane region [38]. β-secretase first cleaves APP at the β site into an N-terminal soluble secreted amyloid precursor protein β (sAPPβ) and a C-terminal fragment C99 containing 99 amino acids. Then, γ-secretase hydrolyzes the C99 fragment in the proximal N-terminal transmembrane region to release an Aβ peptide consisting of 39–43 amino acids. This process is known as the amyloid degradation pathway of APP [38,39]. The non-amyloid degradation pathway of APP is mediated by α- and γ-secretase. At the cell membrane, APP is cleaved by α-secretase to produce an N-terminal soluble secreted amyloid precursor protein α (sAPPα) and a C-terminal fragment C83 containing 83 amino acids, which is subsequently cleaved by γ-secretase to release the APP intracellular domain (APP intracellular domain (AICD) and a non-toxic P3 protein [40,41]. Since the site of action of α-secretase is in the Aβ region, it prevents the production of Aβ.

**Figure 1:**
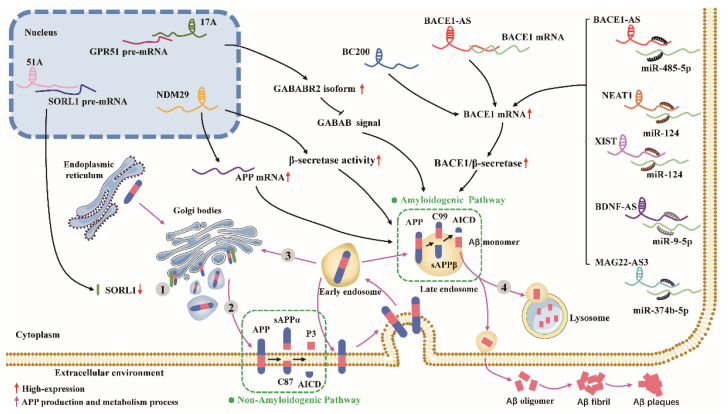
Schematic representation of regulatory mechanisms of lncRNAs during APP processing and Aβ production. lncRNAs are first produced in the endoplasmic reticulum, modified by Golgi processing, and transported to the plasma membrane, where they primarily enter the nonamyloid processing pathway (green box). lncRNAs are internalized from the plasma membrane to form early endonucleosomes, from where they: (1) Can be transported again to the plasma membrane to enter the recycling pathway, (2) Can be returned to the TGN via the reverse-transcriptase-mediated pathway, (3) Can form late intranucleosomes that enter the amyloid processing pathway (green box) or fuse with lysosomes for degradation; the above pathways are indicated by pink arrows. Grey circles marked with numbers 1–4 represent APP processing and Aβ production with the involvement of SORL1. lncRNAs regulate APP processing and Aβ production by a variety of specific mechanisms, including mRNA transcription (NDM29 and BC200), mRNA splicing (51A and 17A), miRNA sponges (BACE-AS, NEAT1, XIST, BDNF-AS, and MAG22-AS), mRNA stability (BACE1-AS), and protein activity (NDM29).

APP is synthesized in the endoplasmic reticulum, processed and modified by the Golgi complex, and transported to the trans-Golgi network (TGN), one of the major sites of Aβ production [42]. APP can be degraded by β- and γ-secretase upon retention in the TGN to produce Aβ. Undegraded APP can be transported to the cell membrane surface via secretory vesicles generated by the TGN, where α-secretase localizes and then mediates its nonamyloid degradation pathway [42]. Unprocessed APP is internalized from the cell surface by interacting with lattice proteins near the cell membrane and adapter protein 2 (AP2) [43]. The internalized APP first forms early nuclear endosomes and is then sorted into the following pathways: (I) A small fraction of the APP molecules re-enters the cell membrane and then enters the nonamyloid processing pathway [44,45]; (II) Some APP molecules are then translocated back to the TGN or form late endosomes and enter the amyloid processing pathway [46]; (III)Another fraction of APP molecules fuses with lysosomes that are degraded [46]. lncRNAs affect the production of Aβ through different mechanisms of action (Figure 1).

### 3.1. lncRNA BACE1-AS

Beta-site amyloid precursor protein cleaving enzyme 1 (BACE1) is a type-I transmembrane aspartic protease consisting of 501 amino acids with a β-secretase role [47]. Although molecular genetic analysis failed to identify any genetic link between BACE1 and familial AD, its expression level and enzymatic activity were enhanced in a few brain samples from patients with early and late-onset AD [48,49]. Moreover, the knockdown of BACE1 in APP transgenic mice reduced the Aβ burden in the mouse brain to a large extent and did not affect the healthy phenotype of the mice [50]. Thus, the expression levels of the BACE1 gene closely regulate the APP processing pathway and play a key role in the pathogenesis of AD.

BACE1 antisense transcript (BACE1-AS) is a conserved long noncoding RNA 2 kb in length, and BACE1 mRNA are two transcripts from the same locus in human chromosome 11 (11q23.3), with BACE1 mRNA transcribed from the sense strand and BACE1-AS from the antisense strand [51]. BACE1-AS structurally contains a 5’ capping and a poly-A tail. BACE1-AS is highly expressed in AD patients as well as in APP transgenic mice, and BACE1-AS regulates the expression levels of BACE1 mRNA and protein both in vitro and in vivo. BACE1-AS, through different modes of action, not only enhances the stability of BACE1 mRNA but also promotes BACE1 mRNA expression, leading to increased Aβ1-42/Aβ1-40 production by APP via the amyloid processing pathway and accelerating the development of AD. BACE1-AS can pair with BACE1 mRNA to form RNA duplexes, leading to structural changes and enhanced stability of BACE1 mRNA [51]. BACE1-AS acts as ceRNA, masking the binding site of miR-485-5p on BACE1 mRNA, thus inhibiting the repressive effect of miR-485-5p on BACE1 mRNA and promoting the expression of BACE1 mRNA [52,53]. Short interfering RNA (siRNA)-mediated silencing of BACE1-AS expression in human SH-SY5Y cells in vitro attenuated the ability of BACE1 to cleave APP and reduced the production of Aβ1-42 [54]. Intracranial injection of siRNA BACE1-AS into AD model mice not only downregulated BACE1 protein levels but also significantly reduced insoluble Aβ production and improved learning and memory abilities in mice [55]. Notably, exogenous Aβ1-42 can promote BACE1-AS expression in neurons, increase the stability of BACE1 mRNA, and produce additional Aβ1-42, thus forming a positive feedback loop to promote AD development [52]. Silencing of BACE1-AS regulated autophagy through the miR-214-3p/ATG5 signaling axis and attenuated Aβ-induced neuronal injury [56]. Ge et al. found that BACE1-AS knockdown also protected neuronal cells from Aβ25-35 damage by targeting miR-132-3p [57]. These findings suggest that lncRNA BACE1-AS is a promising target for the treatment of AD.

### 3.2. lncRNA MAGI2-AS3

Membrane-associated guanylate kinase inverted 2 is a novel lncRNA transcribed from chromosome 7q21.11. Its expression is usually concentrated in the nucleus in SK-N-SH cells [58,59]. MAGI2-AS3 are thought to be cell viability regulators in various diseases [60,61]. MiR-374b-5p plays a vital function in neurogenesis by promoting the proliferation and differentiation of neural stem cells [62]. Zhang et al. analyzed the expressions of MAGI2-AS3 and miR-374b-5p in the serum samples of AD patients [14]. They found that the expression of MAGI2-AS3 was significantly upregulated in AD patients’ serum compared with healthy controls, whereas the miR-374b-5p levels were downregulated. Moreover, the expression level of MAGI2-AS3 positively correlated with the disease severity in AD patients, whereas the opposite was true for miR-374b-5p. Importantly, the same results were obtained for MAGI2-AS3 and miR-374b-5p expression in AD model cells constructed with Aβ. These findings suggest that serum MAGI2-AS3 may serve as a diagnostic marker for AD. Zhang et al. found that MAGI2-AS3 indirectly regulates BACE1 expression by targeting miR-374b-5p, and MAGI2-AS3 inhibition could attenuate Aβ25-35-induced neurotoxicity and neuroinflammation [14]. This suggests that other lncRNAs regulate the expression of BACE1 in addition to BACE-AS. The reversal of Aβ-induced neurotoxicity by MAGI2-AS3 knockdown may be achieved by less Aβ production.

### 3.3. lncRNA BC200

Brain cytoplasmic 200 (BC200), also known as brain cytoplasmic RNA1 (BCYRN1), is a long noncoding RNA containing 200 nucleotides, transcribed by RNA polymerase III and located on human chromosome 2p16 [63]. lncRNA BC200 is specifically expressed in the cytoplasm of neurons [64]. Low expression levels of lncRNA BC200 can be detected in normal tissues in general, whereas in the brain of AD patients, especially in brain regions closely associated with clinical symptoms, such as the hippocampus, the expression level of BC200 is significantly elevated. Importantly, its elevation correlates with the severity of the disease [65].

BC200 was significantly increased in AD cell models constructed using Aβ1-42; by downregulating BC200, it was able to suppress BACE1 mRNA and protein expression as well as rescue Aβ1-42-mediated cell activity reduction and cell apoptosis [66]. BC200 may be a potent positive regulator of BACE1 in AD cells and promote Aβ production. A lncRNA BC1, a mouse sequence homologous to human lncRNA BC200, normally binds to the APP mRNA coding region and inhibits APP mRNA translation. In AD mice, lncRNA BC1 expression is elevated; its ability to target the N-terminal region of FMRP disrupts the binding of FMRP to the APP mRNA coding region, thereby inducing APP translation. When BC1 is downregulated or organized, association with BC1-FMRP suppresses APP translation, thereby blocking the aggregation of Aβ in the brain and preventing memory and spatial learning impairment in AD mice [67]. The specific mechanism of action of BC200 in regulating BACE1 is unknown and needs further investigation.

### 3.4. lncRNA 17A

The next lncRNA is 17A, which is 159 nt in length and transcribed from chromosome 9q22.33 by RNA polymerase III [68]. It is not strictly a lncRNA in terms of length, but it maps onto intron 3 of the human G protein-coupled receptor 51 (GPR51 and GABAB2 receptor) gene in an antisense conformation. Recently, 17A was found to be highly expressed in the hippocampus and cerebral cortex of AD patients and closely associated with clinical symptoms [69,70,71], which suggests that it may play a direct or indirect role in the pathogenesis of AD.

lncRNA 17A can mask the recognition site of the trans-acting splicing regulator on GPR51 pre-mRNA by RNA pairing. This selective splicing leads to increased translation of the anti-gamma amino butyric acid B receptor 2 (GABABR2) subtype of the receptor, thereby eliminating GABA B2 intracellular signaling (i.e., inhibition of cAMP accumulation and activation of K(+) channels). In turn, this promotes Aβ production and increases the Aβ1-42/Aβ1-40 ratio [68]. Inflammatory stimuli can promote 17A synthesis, and Aβ can lead to an inflammatory environment in the brain [72], which may suggest that Aβ can further promote 17A synthesis by promoting an inflammatory response, forming a positive feedback mechanism.

### 3.5. LncRNA 51A

The sortilin-related receptor L1 (SORL1, commonly referred to as SORLA or LR11) gene is located on human chromosome 11q23.2-q24.2. Its encoded protein, SORL1, is a functionally unknown transmembrane neuronal sorting protein, which is specifically and abundantly expressed in neurons [73,74,75]. Katrin et al. found that in the cortical and hippocampal neurons of AD patients, SORL1 expression levels were significantly reduced [76,77]. Ma et al. also reported that the expression of SORL1 is reduced in the cerebrospinal fluid (CSF) of AD patients [78]. Furthermore, BACE1 activity detected in the CSF positively correlated with the SORL1 concentration [79]. These findings suggest a potential role for SORL1 in the pathogenesis of AD. SORL1 plays a key role in the transport and processing of APP and Aβ. SORL1 binds to APP, retaining it in the TGN and preventing APP from forming homodimers, the preferred substrate for β-secretase [80]. SORL1 prevents the transfer of APP from the TGN to the cell membrane [81]. SORL1 interacts with different cytoplasmic adapters to direct internalized APP into the TGN, thus limiting the delivery of APP to the endocytic region that facilitates amyloid processing. SORL1 interacts with Aβ and promotes lysosomal sorting of Aβ for its intracellular degradation [82]. SORL1 downregulation enhances the amyloidogenic process of APP and significantly increases the risk of sporadic Alzheimer′s disease (SAD) and familial Alzheimer′s disease (FAD) [83,84]. SORL1 has been identified as an important gene for AD.

lncRNA 51A is approximately 300 nucleotides in length and is reverse transcribed from the antisense strand of intron 1 of the SORL1 gene by RNA polymerase III. In contrast to SORL1, lncRNA 51A levels are significantly upregulated in the brains of AD patients [85]. Mechanistically, lncRNA 51A binds to the splice site of SORL1 pre-mRNA via base-pairing, resulting in a splice shift that reduces the expression of the canonical variant A [85]. Thus, SORL1, a protective gene for AD, and lncRNA 51A may play an important role in AD by suppressing the expression of canonical variant A of SORL1.

### 3.6. lncRNA NDM29

Neuroblastoma differentiation marker 29 (NDM29), a lncRNA transcribed by RNA polymerase III, was isolated in the context of the search for small nuclear (sn) RNA-like promoters in the human genome [86,87]. It is located in the chromosome 11p15.3 region, associated with oncogenic activity [88,89], and is an important factor driving the differentiation of neuroblastoma (NB) cells to a nonmalignant neuron-like phenotype [90,91]. A comparison of NDM29 expression in the cerebral cortex of AD patients and non-diseased control individuals revealed that NDM29 can be synthesized in normal human brains but is expressed more in the brains of AD patients [92]. Massone et al. further explored whether NDM29 expression affects the production of Aβ in neuroblastoma cells [92]. The results showed that NDM29 significantly increased APP mRNA and protein levels, while increasing β- and γ-secretase activities, thus placing APP in the amyloid processing pathway and not only causing a general increase in total amyloid secretion but also an elevated ratio of Aβ x-42 and Aβ x-40. Notably, as with lncRNA 17A, proinflammatory molecules, such as interleukin 1α (IL-1α) and tumor necrosis factor α (TNF-α), can promote the synthesis of NDM29, whereas anti-inflammatory drugs (diclofenac) can inhibit the synthesis of lncRNA NDM29.

### 3.7. lncRNA BDNF-AS

Brain-derived neurotrophic factor (BDNF) is the most widely distributed neurotrophic growth factor in the central nervous system. It is essential for neuronal development and survival, neurite growth and differentiation, synaptic plasticity, and neurotransmitter release [93,94,95,96,97]. Mice with a knockdown of BDNF died in the second week after birth [98]. In AD patients, the expression of mRNA and the protein level of BDNF are severely decreased in the hippocampus, temporal lobe, frontal lobe, and parietal cortex [99,100,101,102,103,104,105,106]. In addition, serum BDNF concentrations are consistently lower in AD patients compared with healthy elderly subjects [107,108,109,110,111] and correlate with MMSE [112,113,114]. Multiple lines of evidence suggest that Aβ can contribute to cognitive dysfunction and memory loss by downregulating BDNF expression [115,116,117]. Aβ reduces BDNF levels by decreasing phosphorylated cyclic adenosine monophosphate (cAMP) response element binding protein (CREB). In primary cultured neurons, BDNF pretreatment showed a potential protective effect against Aβ-induced neurotoxicity [118]. Thus, BDNF plays a key role in Aβ-induced synaptic damage in neuronal cells and cognitive dysfunction in AD patients.

The BDNF antisense transcript (BDNF-AS) is a conserved lncRNA, and BDNF mRNA are two transcripts at the same locus in human chromosome 11 (11q23.3) [119]. The transcription start site (TSS) of human BDNF-AS is located approximately 200 kb downstream of the BDNF promoter. Transcription from this site generates 16–25 splice variants of BDNF-AS with 6–8 exons. Exon 4 is common to all these variants; exon 5, containing 225 nucleotides, is fully complementary to BDNF mRNA. Thus, BDNF-AS has the potential to form an in vivo RNA–RNA duplex with BDNF mRNA through the overlap of 225 complementary nucleotides [120]. However, unlike BACE1-AS, which enhances the stability of BACE1, BDNF-AS does not affect the stability of BDNF. Specifically, BDNF-AS recruits Ezh2 (one of the components of polycomb repressive complex 2) to the BDNF promoter region to catalyze the trimethylation of histone H3-lysine 27 (H3K27met3), thereby repressing the transcription of BDNF mRNA [120]. BDNF-AS stimulates Aβ production by competitively binding miR-9-5p to promote the expression of BACE1 [121]. Guo et al. exposed PC12 cells to Aβ25-35 to establish AD model cells [122]. They found that Aβ25-35 significantly increased BDNF-AS levels and decreased BDNF levels in PC12 cells, which were accompanied by a decrease in PC12 cell viability and apoptosis induction. However, the silencing of BDNF-AS significantly upregulated the Aβ25-35-induced decrease in BDNF, increased cell viability, and inhibited apoptosis in PC12 cells. Based on these studies, we found that a positive loop forms between Aβ and BDNF-AS, which ultimately inhibits BDNF expression. Therefore, the inhibition of BDNF-AS is a promising strategy to treat AD, specifically by increasing BDNF levels.

## 4. Regulatory Effects of lncRNAs on Aβ Clearance

**Figure 2:**
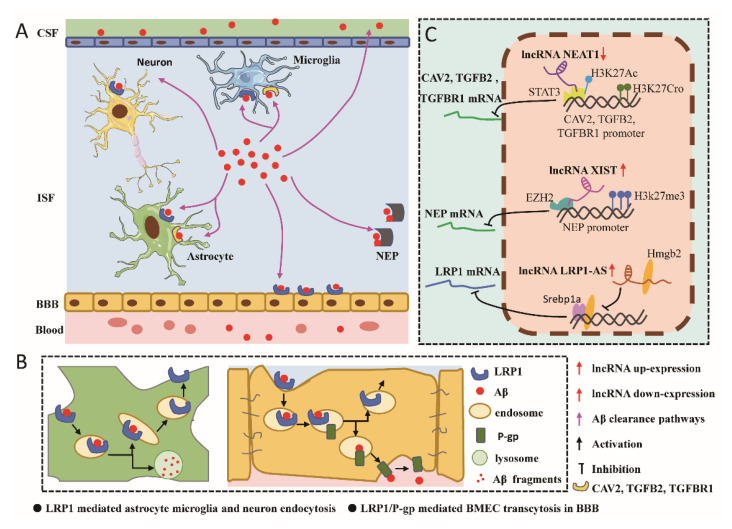
Mechanisms of lncRNA in Aβ clearance pathway. (**A**) Overview of Aβ clearance pathway. (**B**) Schematic representation of LRP1-mediated endocytosis in microglia, astrocytes, and neurons. Schematic representation of LRP1/P-gp-mediated transmigration of brain microvascular endothelial cells in BBB. This process primarily involves Aβ being internalized by LRP1 on brain microvascular endothelial cells in BBB and delivered to P-gp, which then secretes Aβ on the luminal side of BMEC, while LRP1 is recycled to the cell surface. (**C**) Specific mechanisms in the regulation of Aβ clearance by lncRNAs. In early AD, downregulation of lncRNA NEAT1 reduces glial-cell-mediated Aβ clearance through epigenetic histone modifications that inhibit the expression of endocytosis-related genes (CAV2, TGFB2, and TGFBR1). In AD, lncRNA XIST expression is increased, leading to reduced NEP expression via increasing enrichment of EZH2 and H3K27me3 in the NEP promoter region, thereby inhibiting enzymatic clearance of Aβ in cells. lncRNA LRP1-AS expression is increased in AD, and it directly binds to Hmgb2 and inhibits the Hmgb2-enhanced Srebp1a transcriptional activity on LRP1, thereby inhibiting LRP1 expression and thereby reducing glial- and neuronal-mediated Aβ clearance.

In addition to Aβ production, disorders of Aβ clearance are also a major cause of AD pathology. Aβ clearance can be accomplished by a variety of mechanisms: (I) Transport of Aβ from the ISF to the CSF via lymphatic drainage [123]; (II) Endocytosis of microglia, astrocytes, and neurons leading to degradation of Aβ in lysosomes [124,125,126,127]; (III) Transport of Aβ across the blood–brain barrier (BBB) into the circulation [123,128]; (IV) Degradation by extracellular proteases such as neurolysin (NEP), insulin-degrading enzyme (IDE), matrix metalloproteinases 9 (MMP-9), and so on [129]. lncRNAs affect the clearance of Aβ through different mechanisms of action (Figure 2).

### 4.1. lncRNA LRP1-AS

Low-density lipoprotein receptor-related protein 1 (LRP1) belongs to the low-density lipoprotein receptor family and is highly expressed in the brain [130,131]. LRP1 is important in Aβ clearance because it mediates the uptake and degradation of Aβ in astrocytes, microglia, and neurons [124,125,126,127]. Together with ABCB1/P-glycoprotein (P-gp), it coordinates Aβ transmigration across the BBB via cerebrovascular smooth muscle cells [128]. In vitro and in vivo studies confirmed that impairment of LRP1 endocytosis inhibits the brain clearance of Aβ. However, LRP1 not only promotes Aβ clearance via endocytosis but also binds to APP at the cell surface and promotes APP endocytic transport, thereby increasing amyloid processing. Furthermore, the C-terminal transmembrane domain of LRP1 reduces Aβ production by competing with APP for the cleavage site of β- and γ-secretase [130]. Thus, LRP1 has an important dual role in the production and clearance of Aβ. Consequently, we do not know if LRP1 is more inclined to produce Aβ or to scavenge Aβ in vivo. To analyze the net effect of LRP1 on Aβ production and clearance in vivo, Bart Van Gool et al. crossed mice with impaired LRP1 function with a mouse model of AD. They presented exact, in vivo evidence that global impairment of LRP1′s endocytosis function favors the nonamyloidogenic processing of APP due to its reduced internalization and, subsequently, reduced amyloidogenic processing. By inactivation of LRP1, the inhibitory effect on Aβ generation over-rules the simultaneous impaired Aβ clearance, resulting in less extracellular Aβ and reduced plaque deposition in a mouse model of AD.

LRP1-AS is a 1387 nt lncRNA that is a natural antisense transcript of low density lipoprotein receptor-related protein 1 (LRP1), transcribed from the opposite strand of the LRP1 gene and negatively regulates LRP1 expression [132]. Exon 2 of human LRP1-AS contains two short open reading frames (ORFs) of 141 bp (15–155 bp) and 108 bp (226–333 bp). Similarly, exon 2 of mouse LRP1-AS also contains two short ORFs of 120 bp (388 to 507) and 117 bp (675 to 791). Exon 2 of mouse LRP1-AS directly overlaps exons 5 and 6 of LRP1 by 395 bp, whereas exon 2 of human LRP1-AS directly overlaps exon 5 of LRP1 by 119 bp. LRP1/LRP1-AS has remained in a similar location throughout evolution. Yamanaka et al. found that LRP1-AS binds directly to high-mobility group box 2 (Hmgb2) and suppresses Hmgb2-enhanced Srebp1a transcriptional activity on LRP1. The function of LRP1-AS is further regulated by LRP1 mRNA, which can base pair with LRP1-AS to form an RNA duplex, preventing LRP1-AS and Hmgb2 interaction. LRP1-AS short oligonucleotides suppress antisense transcript-Hmgb2 protein interaction and enhance LRP1 expression by increasing Hmgb2 activity [132]. RNA, extracted from the frontal gyrus of AD patients and their age-matched controls, were examined by qRT-PCR, which revealed a decrease in the expression of LRP1 mRNA levels in AD patients and an increase in the levels of LRP1-AS [132]. These implicate LRP1-AS as being involved in the development of AD by repressing the transcription of LRP mRNA.

### 4.2. lncRNA NEAT1

Nuclear abundant transcript 1 (NEAT1) is a cytosolic-enriched lncRNA transcribed from the multiple endocrine neoplasia type 1 (MEN1) gene and is one of the lncRNAs involved in the formation and maintenance of paraspeckles [133]. Spreafico et al. found that NEAT1 expression levels were increased in the temporal cortex and hippocampus of AD patients compared with the controls [134]; thus, the role of NEAT1 in AD pathology has attracted close attention [135,136]. In the brain tissues of the AD mouse model, NEAT1 expression is increased, and miR-124 expression is decreased [85]. NEAT1, as ceRNA, up-regulates BACE1 mRNA and protein levels by regulating the miR-124/BACE1 axis. Increased Aβ, in turn, increases NEAT1 and BACE1 expression and suppresses miR-124 levels, and these effects are reversed by NEAT1 knockdown. This suggests that lncRNA NEAT1 and Aβ are in a mutually reinforcing relationship. A recent study found that NEAT1 expression is suppressed in the early stages of AD and that it inhibits neuroglial cells, mediating Aβ clearance via epigenetic regulation of the expression of endocytosis-related genes (CAV2, TGFB2, and TGFBR1) [137]. The downregulation of NEAT1 inhibits acetyl-CoA generation and the autoacetylation of P300, and then decreases H3K27 acetylation (H3K27Ac) and increases H3K27 crotonylation (H3K27Cro) near the TSS of endocytosis-associated genes, thus suppressing the expression of endocytosis-associated genes [137]. ROR1, one of the components of the Ror family, is specifically involved in neurite extension and neurogenesis and plays a key role in establishing neuronal networks. Ke et al. recently reported that NEAT1 knockdown reduces Aβ-induced neuronal damage and phosphorylated tau protein levels via the miR-146a-5p/34a-5p/ ROR1 pathway [135]. This suggests that the expression level of lncRNA NEAT1 is spatiotemporally specific, with decreased expression in the early stage of AD, leading to reduced Aβ clearance; with the accumulation of Aβ, lncRNA NEAT1 expression increases, further promoting the production of Aβ, thus forming a vicious cycle leading to the development of AD.

### 4.3. lncRNA XIST

X-inactive specific transcript (XIST) is a 17–19 kb long lncRNA transcribed from the XIST gene located on the X chromosome (Xq13.2). The XIST gene is a component of the XIC, the X chromosome inactivation center. XIST mediates the silencing of gene transcription on the X chromosome by recruiting specific protein complexes and plays a key role in X chromosome inactivation [138,139]. lncRNA XIST plays an essential role in AD [140].

Neurolysin (NEP), encoded by the NEP or MME gene, is an important peptidase involved in the degradation of β-amyloid protein [141]. In pathological conditions of AD, the expression level and activity of NEP decrease, and specific polymorphisms of the NEP gene increase the risk of AD [142]. As an essential neuropeptide and amyloid-degrading enzyme, NEP has become a therapeutic target for AD [143]. Recently, Yan et al. found that lncRNA XIST expression was increased in AD mice and cellular models and negatively correlated with NEP expression. The knockdown of lncRNA XIST reduced the enrichment of EZH2 and H3K27me3 in the NEP promoter region, which led to an increase in NEP expression and thus facilitated the enzymatic degradation of Aβ in cells [144]. Du et al. found that lncRNA XIST was significantly upregulated in H_2_O_2_-induced AD mouse models and N2a cells and was involved in the development of AD by positively regulating BACE1 expression through interaction with miR-124 [145]. These findings suggest that lncRNA XIST plays a key role in the production and clearance of Aβ, but the ultimate result is the promotion of Aβ accumulation.

## 5. Regulatory Effects of lncRNAs on Aβ-Induced Neurotoxicity

### 5.1. lncRNA ATB

lncRNA ATB, located on chromosome 14, is one of the most important regulatory RNAs and is overexpressed in many human cancers [146]. lncRNA ATB expression was significantly increased in the blood and CSF of AD patients and was consistently altered in an Aβ-induced AD cell model [147]. Knockdown of lncRNA-ATB suppressed Aβ-induced neurotoxicities, such as cell viability reduction, apoptosis, cytotoxicity, and oxidative stress. This was achieved through an ATB-mediated miR-200 sponge mechanism, which selectively targets zinc finger gene 217 (ZNF217) in PC12 cells. ZNF217, a member of the Krüppel-like family, is a transcription factor that plays a key regulatory role in many diseases. However, the mechanism by which it promotes Aβ-induced neurological damage in AD is currently unknown and needs further exploration [148].

### 5.2. lncRNA RPPH1

The ribonuclease P RNA component H1 (RPPH1), as the RNA component of ribonuclease P ribonucleoprotein, cleaves tRNA precursor molecules to produce mature tRNA [149]. In primary cultured hippocampal pyramidal neurons overexpression of lncRNA RPPH1 leads to an increase in dendritic spine density, whereas lncRNA RPPH1 knockdown has the opposite effect. Additionally, lncRNA RPPH1 regulates CDC42 expression by targeting miR-326 to increase hippocampal neuronal dendritic spines [150]. lncRNA RPPH1 was upregulated in cortical tissues from APP/PS1 double-transgenic mice [151,152]. In AD pathology, lncRNA RPPH1 enables neuroprotection through two distinct ceRNA axes: RPPH1/miR-326/Pyruvate kinase M2 (PKM2) [151] and RPPH1/miR-122/WNT1 [153]. RPPH1 attenuates Aβ25-35-induced cell viability reduction, endoplasmic reticulum stress, and apoptosis in SH-SY5Y cells [151]. RPPH1, as a ceRNA, targets and regulates miR-326 to increase the expression of PKM2. PKM2 affects cell death and apoptosis by regulating glycolytic metabolism. Wnt signaling pathway activation can block Aβ-dependent neurotoxicity, and dysfunction of the Wnt/β-catenin signaling pathway is closely associated with Aβ toxicity and BBB breakdown in AD [153,154]. Ran et al. found that lncRNA RPPH1 protected Aβ-induced neuronal injury in SK-N-SH cells via targeting miR-122 and activating downstream Wnt/β-catenin signaling [153]. These findings suggest that lncRNAs play an important part in suppressing Aβ-induced neurotoxicity.

### 5.3. lncRNA H19

lncRNA H19 is a conserved lncRNA, transcribed from chromosome 11p15, and is located in both the nucleus and cytoplasm of cells [155,156]. It is 2.3 kb long and consists of five exons and four small introns by splicing, 5′ methyl capping, and 3′ polyadenylation. lncRNA H19 was highly expressed in the hippocampal group of APP/PS1 double-transgenic mice [157]. To investigate the role of lncRNA H19 in AD, Zhang et al. found that lncRNA H19 expression was increased in Aβ-induced AD model cells and was mainly distributed in the cytoplasm. The suppression of H19 expression could inhibit Aβ-induced cell apoptosis, viability reduction, and oxidative stress through the miR-124/MGB1 axis [158].

### 5.4. lncRNA SNHG1

Small nucleolar RNA host gene 1 (SNHG1) is a newly discovered lncRNA whose coding gene is located at 11q12.3. It is highly expressed in many tumor tissues and is considered an oncogene regulating cancer progression [159]. The level of SNHG1 was also significantly elevated in Aβ-treated SK-N-SH cells. lncRNA SNHG1 knockdown attenuates the effect of Aβ on cell viability, apoptosis, neuroinflammation, and oxidative stress. This is achieved by targeting ZNF217, a factor with an essential role in Aβ-induced neurotoxicity, via a mechanism of SNHG1-mediated miR-361-3p sponge, which selectively targets the untranslated region of ZNF217 [160]. Mechanistically, Wang et al. also demonstrated the involvement of SNHG1 as a ceRNA in the pathological process of AD. Kringle-containing transmembrane protein 1 (KREMEN1) is a transmembrane receptor that has an intrinsic proapoptotic activity [161]. The knockdown of SNHG1 enhanced the inhibitory effect of miR-137 on KREMEN1 expression and subsequently attenuated Aβ25-35-induced neuronal damage.

### 5.5. lncRNA WT1-

Wilms tumor 1 antisense RNA(WT1-AS) is transcribed from the intron region of WT1 [162]. In tumor tissues, WT1-AS expression is regulated by methylation and aberrant splicing, and the function of WT1-AS is highly tissue- and cell-specific and closely associated with the development of a variety of tumors [163]. Recently, by analyzing the WT1-AS expression profile in the GSE4757 dataset, WT1-AS expression was found to be significantly reduced in AD; this change was also verified in the hippocampal tissue of AD mice [164]. This suggests a possible role for WT1-AS in AD. Wang et al. constructed an in vitro cell model of AD by treating SH-SY5Y with Aβ25-35 and found that WT1-AS was significantly reduced in AD model cells and was mainly expressed in the nucleus. WT1-AS could inhibit Aβ-induced apoptosis and oxidative stress injury. Additional mechanistic studies revealed that WT1-AS could negatively regulate WT1, which could directly target the promoter region of miR-375 to promote its expression, whereas miR-375 could bind to SIX4 to inhibit its expression. Therefore, lncRNA WT1-AS alleviates Aβ-induced neuron injury and apoptosis by regulating WT1 to inhibit the miR-375/SIX4 axis [164].

### 5.6. lncRNA EBF3-AS

Early B cell factor 3 antisense RNA (EBF3-AS) is a lncRNA containing two exons 842 nt in length, transcribed from the opposite strand of the protein-coding gene early B cell factor 3 (EBF3) on chromosome 10. Magistri et al. used RNA sequencing to find that the expression of EBF3-AS was highly differential and abundant in the brain of late-onset AD patients compared to the controls [165]. Therefore, EBF3-AS was hypothesized to be involved in regulating the development of AD. Further examination of the expression of EBF3-AS in the hippocampus of AD model mice revealed that the expression of EBF3-AS was upregulated in the hippocampal tissue of APP/PS1 mice compared with C57BL/6 mice, and the mRNA and protein expression of EBF3 was upregulated together with the expression of EBF3-AS, suggesting that EBF3 may be a downstream target gene of EBF3-AS. Similarly, the expression level of lncRNA EBF3-AS was upregulated in Aβ-induced AD model cells, and lncRNA EBF3-AS knockdown alleviated the Aβ-induced decrease in cell activity and apoptosis by downregulating EBF3 [166].

### 5.7. lncRNA SNHG19

In 2019, Cao et al. used gene array datasets and bioinformatics analysis to identify age- and sex-related differentially expressed lncRNAs in the AD human brain. They found for the first time that the lncRNA small nucleolar RNA host gene 19 (SNHG19) was differentially expressed in the AD human brain. Moreover, the expression of lncRNA SNHG19 was positively correlated with the Braak stage of AD [13]. Further study of its role in AD revealed that SNHG19 expression was dose-dependently upregulated in Aβ-induced SH-SY5Y cells. TNFAIP1 mRNA levels are significantly increased in the transgenic *C. elegans* model of AD, APP/PS1 transgenic mice, and postmortem brain tissue of AD patients [167]. TNFAIP1 promotes neurotoxicity by inhibiting AKT/CREB signaling [168,169]. SNHG19 knockdown rescued Aβ25-35-induced SH-SY5Y cytotoxicity via regulating the miR-137/TNFAIP1 axis [170].

### 5.8. lncRNA SOX21-AS1

SRY-Box 21 antisense RNA 1 (SOX21-AS1) is a 2986 bp lncRNA that shares a bidirectional promoter with SOX21 at human chromosome 13q32.1 [171]. Zhang et al. performed a microarray analysis of data from AD chip GSE4757 and found that SOX21-AS1 was highly expressed in AD. They constructed the AD model by injecting 1 μL of Aβ1-40 with microaggregated peptide into the hippocampal region of mice. Frizzled protein 3/5 (FZD3/5) is an essential receptor for the Wnt signaling pathway and participates in the development of the central nervous system. They found that knockdown of SOX21-AS1 upregulated the expression of FZD3/5 and activated the Wnt signaling pathway, which in turn improved learning and memory in AD mice and inhibited oxidative stress, apoptosis, and Aβ expression levels in hippocampal neurons [172]. Xu et al. found that the expression level of SOX21-AS1 was significantly elevated in an in vitro cell model of AD constructed with Aβ in a concentration- and time-dependent manner [173]. Knockdown of SOX21-AS1 attenuated Aβ-induced cell viability reduction, apoptosis, and hyperphosphorylation of tau protein by sponging miR-107 [173]. The PI3K/AKT signaling pathway has neuroprotective effects in AD by regulating multiple substrates. SOX21-AS1 knockdown also attenuates Aβ-dependent neuronal cell damage by promoting the miR-132/PI3K/AKT pathway [174]. These findings suggest that lncRNAs can moderate pathological changes in AD through multiple signaling pathways.

### 5.9. lncRNA SNHG7

The small nucleolar RNA host gene (SNHG7) is a 2157 bp long lncRNA transcribed from chromosome 9q34.3, first reported by Chaudhry in 2013 [175]. Disruption of the BBB is considered a severe pathological hallmark of AD development. Aβ deposition induces the hyperpermeability of the BBB by disrupting tight junction (TJ) proteins formed by endothelial cells (ECs) [176,177]. SNHG7 and the trans-activation response RNA-binding protein 2 (TARBP2) are upregulated in ECs incubated with Aβ1-42, and TARBP2 directly binds to SNHG7 to increase its stability. The nuclear factor of activated T cells isoform c3 (NFATC3) plays a role in endothelial cell biology [178,179]. SNHG7 suppression promotes TJ-related protein expression through miR-17-5p/NFATC3 axis and protects the BBB [180].

### 5.10. lncRNA ANRIL

lncRNA antisense noncoding RNA at the INK4 locus (ANRIL), also known as CDKN2B-AS1 or CDKN2B-AS1, is 3.8 kb in length and consists of 19 exons, which are transcribed by RNA polymerase II from the opposite direction of the INK4/ARF gene cluster on chromosome 9p21 [181]. ANRIL is found in many diseases associated with inflammation and neurological dysfunction. Feng et al. investigated the role of ANRIL in AD biology. Specifically, ANRIL expression was elevated in AD model cells, and ANRIL suppression ameliorated Aβ-induced neurotoxicity, such as cell activity reduction, apoptosis, and neurite growth inhibition, by targeting miR-25a. Based on these results, ANRIL may be a notable marker and therapeutic target in AD [182].

### 5.11. lncRNA MALAT1

Metastasis-associated lung adenocarcinoma transcript-1 (MALAT1), also named NEAT2, is a well-conserved lncRNA, transcribed on chromosome 11q13.1. It belongs to the intergenic noncoding RNA, composed of 8828 nucleotides [183,184]. MALAT1, like the housekeeping proteins GAPDH and β-actin, is abundantly expressed in the cell and preferentially localized in the nucleus [185]. MALAT1 is also highly abundant in neurons and closely associated with neuronal synapse formation [186]. In addition, its anti-inflammatory and neuroprotective effects have been studied in various diseases, such as multiple sclerosis [187]. Compared to the controls, no significant difference was found in the expression level of lncRNA NALAT1 in the brain of AD patients [134], but low levels of MALAT1 were detected in the CSF [188]. The expression of MALAT1 was also reduced in Aβ1-42-treated primary cortical neurons [189]. Similarly, Li et al. found reduced MALAT1 expression in the hippocampal tissue of AD mice [190]. These findings show that MALAT1 is reduced in the AD brain. However, this is different from the results reported by Spreafico et al., where MALAT1 interacts with miR125b to inhibit Aβ-induced apoptosis and inflammation in neurons, while promoting neurite protrusion growth. PTGS2, CDK5, and FQXQ1 are all possible downstream targets of miR-125b [189]. The PI3K/AKT signaling pathway plays an important neuroprotective role in AD, but is always poorly activated in AD [191]. A novel ceRNA network involving MALAT1/miR-30b/CNR1 can increase neuronal viability and reduce neuronal damage by Aβ in AD cells and animal models in which the PI3K/AKT signaling pathway is activated [190]. A recent study again validated that MALAT1 is significantly reduced in APP/PS1 mice, in cellular models of Aβ constructs, and even in the brains of AD patients. This gives us greater confidence to explore the role of MALAT1 in AD further [192]. Another ceRNA regulatory axis of MALAT1 was identified by Chanda et al. MALAT1/miR-200a/26a/26b/EPHA2 axis overexpression confers protection against Aβ1–42 cytotoxicity through its downstream effectors CREB, p38, and synaptophysin [192]. These findings suggest that the ceRNA regulatory mechanism of MALAT1 may be an important strategy for controlling the disease in the context of AD pathophysiology.

## 6. Conclusions

**Figure 3:**
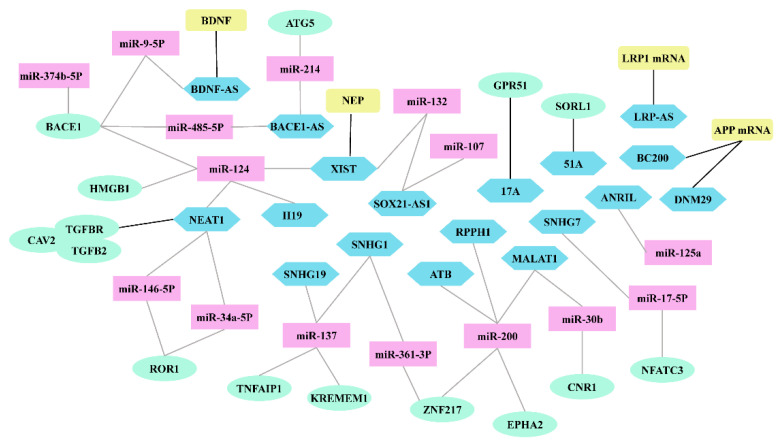
Complexity and interactions of lncRNAs in Aβ cascade hypothesis. Gray lines represent the ceRNA axis of lncRNAs. Black lines represent other mechanisms of action of lncRNAs, including mRNA transcription (NDM29, BC200, and LRP-AS), mRNA splicing (51A and 17A), and histone modification (NEAT1, XIST, and BDNF-AS).

Through high-throughput, whole-transcriptome analysis, researchers have identified many differentially expressed lncRNAs in the brains of AD patients, thereby providing important insights into the biological and clinical relevance of lncRNAs in AD. hese differentially expressed lncRNAs interact with different molecules to form complex functional networks during the Aβ cascade hypothesis (Figure 3). Dysregulation of Aβ homeostasis, leading to Aβ accumulation, is the most important trigger of AD [3,4,5]. lncRNAs regulate the expression of AD-related genes through various mechanisms to play a key role in Aβ production, clearance, and its induced neurotoxicity (Table 1). As gene regulation research continues and more biological approaches emerge, the precise biological functions and molecular mechanisms of lncRNAs will be further elucidated, which will not only deepen the understanding of the pathological mechanisms of AD but also provide more precise theoretical guidance for the diagnosis and treatment of AD. The high tissue specificity and spatiotemporal specificity of lncRNAs make them particularly attractive as diagnostic biomarkers and specific therapeutic targets [193]. Currently, the diagnosis of AD relies on clinical symptoms, cerebrospinal fluid Aβ42, Aβ40, and amyloid PET-CT when Aβ has already been deposited in the brain, and the disease is usually in the middle to late stages. This is one of the reasons why drugs targeting Aβ have repeatedly failed. This diagnosis is only qualitative and does not achieve early disease prediction or staging. The expression of many lncRNAs changes incrementally, detrimentally, or even inversely during AD disease progression, suggesting that lncRNAs may be ideal biomarkers for AD. Additionally, lncRNAs act as functional molecules and their expression may be a more accurate indicator of disease status. lncRNAs such as lncRNA BACE1-AS have shown potential as AD biomarkers [36]. Therefore, we need to further explore the role of lncRNAs in AD diagnosis to provide a theoretical basis for early diagnosis and disease staging of AD. An antisense drug, nusinersen, has been approved for the treatment of spinal muscular atrophy. Significantly, when given to symptomatic patients, nusinersen not only improves disease symptoms but also slows disease progression. Based on the early success of nusinersen, antisense drugs offer remarkable promise as treatments for neurological disorders [194]. Antisense oligonucleotide (ASO)-based lncRNA knockdown approaches may be an innovative therapeutic strategy for the treatment of AD. Aβ accumulation in AD can be reduced by developing specific ASO downregulation lncRNAs, which is effective in AD cell models and animals [54,68,92,132,195]. Therefore, we need to explore the molecular mechanisms and complex interaction networks of lncRNAs further for targeting lncRNAs to regulate Aβ homeostasis as a new therapeutic strategy for AD.

By summarizing the literature, we also found that the target lncRNAs have all been differentially expressed in Aβ-induced animal or cellular models, which raises the question as to whether the abnormal expression of lncRNAs leads to Aβ accumulation or the accumulation of Aβ leads to the abnormal expression of lncRNAs. Aβ is detected in neurons in vivo, and endogenous Aβ42 or exogenously added Aβ42 taken up by cells can be transferred to the nucleus and is always present in low amounts in the nucleus, which supports the notion that Aβ begins to accumulate in the nucleus [196]. Aβ42 can interact with specific gene regulatory elements to affect gene expression. Thus, the main deleterious effects in AD pathogenesis may be mediated by the genetic control activity of Aβ42, as it can act as a repressor or activator of gene transcription. Therefore, if nuclear translocation of Aβ can be inhibited, gene activation of lncRNA may be avoided, thus preventing the positive feedback that triggers Aβ accumulation. Therefore, we also need to explore further the nuclear translocation pattern of Aβ or how Aβ binds to target genes, leading to lncRNA transcription.

## Figures and Tables

**Table 1:** Aberrant expression patterns of various lncRNAs involved in Aβ cascade hypothesis.

LncRNA	Level	Regulatory Mode	Mechanism	Effects on AD	Reference
BACE1-AS	up	modulate mRNA stability	pairs with BACE1 mRNA to form RNA duplexes, leading to structural changes and enhanced stability of BACE1 mRNA	promotes Aβ production	[51]
		miRNA sponge	increase the level of BACE1 mRNA by binding to miR-485-5p as a ceRNA		[52]
			acts as a ceRNA by sponging miR-214-3p and regulating ATG5 expression, promoting autophagy-mediated neuronal damage	promotes Aβ-induced neurotoxicity	[56]
			targets miR-132-3p		[57]
MAGI2-AS3	up	miRNA sponge	increases the level of BACE1 mRNA by binding to miR-374b-5p as a ceRNA	promotes Aβ production	[14]
BC200	up	modulate mRNA transcription	increase the level of BACE1 mRNA and protein	promotes Aβ production	[66]
17A	up	splicing modulation	inhibits the transcription of the canonical isoform of GABAB R2 by affecting GPR51 alternative splicing and impairs the GABAB signaling pathway	promotes Aβ production	[68]
NDM29	up	modulate mRNA transcription	increases the level of APP mRNA and protein	promotes Aβ production	[92]
		modulation protein activity	increases β- and γ-secretase activities		[92]
51A	up	splicing modulation	binds to the splice site of SORL1 pre-mRNA via base-pairing, resulting in a splice shift that reduces the expression of the canonical variant A	promotes Aβ production	[85]
BDNF-AS	up	miRNA sponge	increase the level of BACE1 mRNA by binding to miR-9-5p as a ceRNA	promotes Aβ production	[121]
		histone modification	recruits Ezh2 to the BDNF promoter region to catalyze the trimethylation of histone H3-lysine 27 (H3K27met3), repressing transcription of BDNF mRNA	promotes Aβ-induced neurotoxicity	[120]
LRP1-AS	up	Modulate mRNA transcription	binds directly to Hmgb2 and suppresses Hmgb2-enhanced Srebp1a transcriptional activity on LRP1	inhibit Aβ clearance	[132]
NEAT1	up	miRNA sponge	increase the level of BACE1 mRNA by binding to miR-124 as a ceRNA	promote Aβ production	[136]
			protects ROR1 by binding to 146a-5p and 34a-5p as a ceRNA	inhibits Aβ-induced neurotoxicity	[197]
NEAT1	down	histone modification	inhibits acetyl-CoA generation and autoacetylation of P300, and then decreases H3K27Ac and increases H3K27Cro nearby the TSS of endocytosis-associated genes to inhibit endocytosis-associated genes expression	inhibit Aβ clearance	[137]
XIST	up	miRNA sponge	increase the level of BACE1 mRNA by binding to miR-124 as a ceRNA	promote Aβ production	[145]
			targets miR-132	promotes Aβ-induced neurotoxicity	[198]
		histone modification	recruits Ezh2 to the NEP promoter region to catalyzes the trimethylation of H3K27met3, repressing transcription of NEP mRNA	inhibit Aβ clearance	[144]
ATB	up	miRNA sponge	acts as a ceRNA by sponging miR-200 and regulating ZNF217 expression	promotes Aβ-induced neurotoxicity	[147]
RPPH1	up	miRNA sponge	acts as a ceRNA by sponging miR-326 and regulating PKM2 expression	inhibits Aβ-induced neurotoxicity	[151]
			acts as a ceRNA by targeting miR-122 and activating downstream Wnt/β-catenin signaling	promotes Aβ-induced neurotoxicity	[152]
H19	up	miRNA sponge	Acts as a ceRNA by sponging miR-124 and regulating HMGB1 expression	promotes Aβ-induced neurotoxicity	[158]
SNHG1	up	miRNA sponge	Acts as a ceRNA by sponging miR-361-3p and regulating ZNF217 expression	promotes Aβ-induced neurotoxicity	[160]
			Acts as a ceRNA by sponging miR-137and regulating KREMEN1 expression		[161]
WT1-AS	down	-	regulates the transcription factor WT1 to inhibit the miR-375/SIX4 axis	inhibits Aβ-induced neurotoxicity	[164]
EBF3-AS	up	-	negatively regulates EBF3	promotes Aβ-induced neurotoxicity	[166]
SNHG19	up	miRNA sponge	Acts as a ceRNA by sponging miR-137 and regulating TNFAIP1 expression	promotes Aβ-induced neurotoxicity	[170]
SOX21-AS1	up	miRNA sponge	Acts as a ceRNA by sponging the miR-132 axis to regulate PI3K/AKT pathway	promotes Aβ-induced neurotoxicity	[174]
			negatively regulate miR-107		[173]
		-	upregulates the expression of FZD3/5 and activates the Wnt signaling pathway		[172]
SNHG7	up	miRNA sponge	acts as a ceRNA to regulate the miR-17-5p/NFATC3 signaling pathway and to inhibit TJ-related protein expression.	promotes Aβ-induced neurotoxicity	[180]
ANRIL	up	miRNA sponge	targets miR-125a		[182]
MALAT1	down	miRNA sponge	regulates the expression of receptor tyrosine kinase EPHA2 via sponging miR-200a/26a/26b	inhibits Aβ-induced neurotoxicity	[192]
			Acts as a ceRNA by sponging miR-30b and regulated CNR1 expression		[190]

## Data Availability

Not applicable.

## References

[1] Scheltens P., De Strooper B., Kivipelto M., Holstege H., Chételat G., Teunissen C.E., Cummings J., van der Flier W.M. (2021). Alzheimer’s disease. Lancet.

[2] Haass C., Selkoe D. (2022). If amyloid drives Alzheimer disease, why have anti-amyloid therapies not yet slowed cognitive decline?. PLoS Biol..

[3] Karran E., Mercken M., De Strooper B. (2011). The amyloid cascade hypothesis for Alzheimer’s disease: An appraisal for the development of therapeutics. Nat. Rev. Drug Discov..

[4] Hardy J., Selkoe D.J. (2002). The amyloid hypothesis of Alzheimer’s disease: Progress and problems on the road to therapeutics. Science.

[5] Chen G.F., Xu T.H., Yan Y., Zhou Y.R., Jiang Y., Melcher K., Xu H.E. (2017). Amyloid beta: Structure, biology and structure-based therapeutic development. Acta Pharmacol. Sin..

[6] Spence J.D. (2012). Genetics of atherosclerosis: The power of plaque burden and progression: Invited commentary on Dong C, Beecham A, Wang L, Blanton SH, Rundek T, Sacco RL. Follow-Up association study of linkage regions reveals multiple candidate genes for carotid plaque in Dominicans atherosclerosis 223 (1) (2012) 177–183. Atherosclerosis.

[7] ENCODE Project Consortium (2012). An integrated encyclopedia of DNA elements in the human genome. Nature.

[8] Derrien T., Johnson R., Bussotti G., Tanzer A., Djebali S., Tilgner H., Guernec G., Martin D., Merkel A., Knowles D.G. (2012). The GENCODE v7 catalog of human long noncoding RNAs: Analysis of their gene structure, evolution, and expression. Genome Res..

[9] Kapranov P., Cheng J., Dike S., Nix D.A., Duttagupta R., Willingham A.T., Stadler P.F., Hertel J., Hackermüller J., Hofacker I.L. (2007). RNA maps reveal new RNA classes and a possible function for pervasive transcription. Science.

[10] Ponting C.P., Oliver P.L., Reik W. (2009). Evolution and functions of long noncoding RNAs. Cell.

[11] Zhou X., Xu J. (2015). Identification of Alzheimer’s disease-associated long noncoding RNAs. Neurobiol. Aging.

[12] Annese A., Manzari C., Lionetti C., Picardi E. (2018). Whole transcriptome profiling of Late-Onset Alzheimer’s Disease patients provides insights into the molecular changes involved in the disease. Sci. Rep..

[13] Cao M., Li H., Zhao J., Cui J., Hu G. (2019). Identification of age- and gender-associated long noncoding RNAs in the human brain with Alzheimer’s disease. Neurobiol. Aging.

[14] Zhang J., Wang R. (2021). Deregulated lncRNA MAGI2-AS3 in Alzheimer’s disease attenuates amyloid-β induced neurotoxicity and neuroinflammation by sponging miR-374b-5p. Exp. Gerontol..

[15] Birney E., Stamatoyannopoulos J.A., Dutta A., Guigó R., Gingeras T.R., Margulies E.H., Weng Z., Snyder M., Dermitzakis E.T., Thurman R.E. (2007). Identification and analysis of functional elements in 1% of the human genome by the ENCODE pilot project. Nature.

[16] Djebali S., Davis C.A., Merkel A., Dobin A., Lassmann T., Mortazavi A., Tanzer A., Lagarde J., Lin W., Schlesinger F. (2012). Landscape of transcription in human cells. Nature.

[17] Iyer M.K., Niknafs Y.S., Malik R., Singhal U., Sahu A., Hosono Y., Barrette T.R., Prensner J.R., Evans J.R., Zhao S. (2015). The landscape of long noncoding RNAs in the human transcriptome. Nat. Genet..

[18] St Laurent G., Wahlestedt C., Kapranov P. (2015). The Landscape of long noncoding RNA classification. Trends Genet. TIG.

[19] Kopp F., Mendell J.T. (2018). Functional Classification and Experimental Dissection of Long Noncoding RNAs. Cell.

[20] Li L., Zhuang Y., Zhao X., Li X. (2018). Long Non-coding RNA in Neuronal Development and Neurological Disorders. Front. Genet..

[21] Lanzafame M., Bianco G., Terracciano L.M., Ng C.K.Y. (2018). The Role of Long Non-Coding RNAs in Hepatocarcinogenesis. Int. J. Mol. Sci..

[22] Mukherjee N., Calviello L., Hirsekorn A., de Pretis S., Pelizzola M., Ohler U. (2017). Integrative classification of human coding and noncoding genes through RNA metabolism profiles. Nat. Struct. Mol. Biol..

[23] Cortini F., Roma F., Villa C. (2019). Emerging roles of long non-coding RNAs in the pathogenesis of Alzheimer’s disease. Ageing Res. Rev..

[24] Clark M.B., Johnston R.L., Inostroza-Ponta M., Fox A.H., Fortini E., Moscato P., Dinger M.E., Mattick J.S. (2012). Genome-wide analysis of long noncoding RNA stability. Genome Res..

[25] Beaulieu Y.B., Kleinman C.L., Landry-Voyer A.M., Majewski J., Bachand F. (2012). Polyadenylation-dependent control of long noncoding RNA expression by the poly(A)-binding protein nuclear 1. PLoS Genet..

[26] Shi K., Liu T., Fu H. (2021). Genome-wide analysis of lncRNA stability in human. PLoS Comput. Biol..

[27] Brown J.A., Valenstein M.L., Yario T.A., Tycowski K.T., Steitz J.A. (2012). Formation of triple-helical structures by the 3’-end sequences of MALAT1 and MENβ noncoding RNAs. Proc. Natl. Acad. Sci. USA.

[28] Wilusz J.E., JnBaptiste C.K., Lu L.Y., Kuhn C.D., Joshua-Tor L., Sharp P.A. (2012). A triple helix stabilizes the 3’ ends of long noncoding RNAs that lack poly(A) tails. Genes Dev..

[29] Hezroni H., Koppstein D., Schwartz M.G., Avrutin A., Bartel D.P., Ulitsky I. (2015). Principles of long noncoding RNA evolution derived from direct comparison of transcriptomes in 17 species. Cell Rep..

[30] Amaral P.P., Leonardi T., Han N., Viré E., Gascoigne D.K., Arias-Carrasco R., Büscher M., Pandolfini L., Zhang A., Pluchino S. (2018). Genomic positional conservation identifies topological anchor point RNAs linked to developmental loci. Genome Biol..

[31] Guo C.J., Ma X.K., Xing Y.H., Zheng C.C., Xu Y.F., Shan L., Zhang J., Wang S., Wang Y., Carmichael G.G. (2020). Distinct Processing of lncRNAs Contributes to Non-conserved Functions in Stem Cells. Cell.

[32] Huarte M., Guttman M., Feldser D., Garber M., Koziol M.J., Kenzelmann-Broz D., Khalil A.M., Zuk O., Amit I., Rabani M. (2010). A large intergenic noncoding RNA induced by p53 mediates global gene repression in the p53 response. Cell.

[33] Dimitrova N., Zamudio J.R., Jong R.M., Soukup D., Resnick R., Sarma K., Ward A.J., Raj A., Lee J.T., Sharp P.A. (2014). LincRNA-p21 activates p21 in cis to promote Polycomb target gene expression and to enforce the G1/S checkpoint. Mol. Cell.

[34] Szymanski M., Barciszewska M.Z., Erdmann V.A., Barciszewski J. (2005). A new frontier for molecular medicine: Noncoding RNAs. Biochim. Biophys. Acta.

[35] Lauretti E., Dabrowski K., Praticò D. (2021). The neurobiology of non-coding RNAs and Alzheimer’s disease pathogenesis: Pathways, mechanisms and translational opportunities. Ageing Res. Rev..

[36] Feng L., Liao Y.T., He J.C., Xie C.L., Chen S.Y., Fan H.H., Su Z.P., Wang Z. (2018). Plasma long non-coding RNA BACE1 as a novel biomarker for diagnosis of Alzheimer disease. BMC Neurol..

[37] Amidfar M., de Oliveira J., Kucharska E., Budni J., Kim Y.K. (2020). The role of CREB and BDNF in neurobiology and treatment of Alzheimer’s disease. Life Sci..

[38] Goate A., Chartier-Harlin M.C., Mullan M., Brown J., Crawford F., Fidani L., Giuffra L., Haynes A., Irving N., James L. (1991). Segregation of a missense mutation in the amyloid precursor protein gene with familial Alzheimer’s disease. Nature.

[39] Sala Frigerio C., De Strooper B. (2016). Alzheimer’s Disease Mechanisms and Emerging Roads to Novel Therapeutics. Annu. Rev. Neurosci..

[40] Tanzi R.E., Bertram L. (2005). Twenty years of the Alzheimer’s disease amyloid hypothesis: A genetic perspective. Cell.

[41] Lichtenthaler S.F., Haass C. (2004). Amyloid at the cutting edge: Activation of alpha-secretase prevents amyloidogenesis in an Alzheimer disease mouse model. J. Clin. Investig..

[42] O’Brien R.J., Wong P.C. (2011). Amyloid precursor protein processing and Alzheimer’s disease. Annu. Rev. Neurosci..

[43] Boll W., Rapoport I., Brunner C., Modis Y., Prehn S., Kirchhausen T. (2002). The mu2 subunit of the clathrin adaptor AP-2 binds to FDNPVY and YppØ sorting signals at distinct sites. Traffic.

[44] Haass C., Kaether C., Thinakaran G., Sisodia S. (2012). Trafficking and proteolytic processing of APP. Cold Spring Harb. Perspect. Med..

[45] Das U., Wang L., Ganguly A., Saikia J.M., Wagner S.L., Koo E.H., Roy S. (2016). Visualizing APP and BACE-1 approximation in neurons yields insight into the amyloidogenic pathway. Nat. Neurosci..

[46] Willnow T.E., Andersen O.M. (2013). Sorting receptor SORLA—A trafficking path to avoid Alzheimer disease. J. Cell Sci..

[47] Vassar R., Bennett B.D., Babu-Khan S., Kahn S., Mendiaz E.A., Denis P., Teplow D.B., Ross S., Amarante P., Loeloff R. (1999). Beta-secretase cleavage of Alzheimer’s amyloid precursor protein by the transmembrane aspartic protease BACE. Science.

[48] Fukumoto H., Cheung B.S., Hyman B.T., Irizarry M.C. (2002). Beta-secretase protein and activity are increased in the neocortex in Alzheimer disease. Arch. Neurol..

[49] Yang L.B., Lindholm K., Yan R., Citron M., Xia W., Yang X.L., Beach T., Sue L., Wong P., Price D. (2003). Elevated beta-secretase expression and enzymatic activity detected in sporadic Alzheimer disease. Nat. Med..

[50] Luo Y., Bolon B., Kahn S., Bennett B.D., Babu-Khan S., Denis P., Fan W., Kha H., Zhang J., Gong Y. (2001). Mice deficient in BACE1, the Alzheimer’s beta-secretase, have normal phenotype and abolished beta-amyloid generation. Nat. Neurosci..

[51] Faghihi M.A., Modarresi F., Khalil A.M., Wood D.E., Sahagan B.G., Morgan T.E., Finch C.E., St Laurent G., Kenny P.J., Wahlestedt C. (2008). Expression of a noncoding RNA is elevated in Alzheimer’s disease and drives rapid feed-forward regulation of beta-secretase. Nat. Med..

[52] Faghihi M.A., Zhang M., Huang J., Modarresi F., Van der Brug M.P., Nalls M.A., Cookson M.R., St-Laurent G., Wahlestedt C. (2010). Evidence for natural antisense transcript-mediated inhibition of microRNA function. Genome Biol..

[53] Zeng T., Ni H., Yu Y., Zhang M., Wu M., Wang Q., Wang L., Xu S., Xu Z., Xu C. (2019). BACE1-AS prevents BACE1 mRNA degradation through the sequestration of BACE1-targeting miRNAs. J. Chem. Neuroanat..

[54] Liu T., Huang Y., Chen J., Chi H., Yu Z., Wang J., Chen C. (2014). Attenuated ability of BACE1 to cleave the amyloid precursor protein via silencing long noncoding RNA BACE1-AS expression. Mol. Med. Rep..

[55] Singer O., Marr R.A., Rockenstein E., Crews L., Coufal N.G., Gage F.H., Verma I.M., Masliah E. (2005). Targeting BACE1 with siRNAs ameliorates Alzheimer disease neuropathology in a transgenic model. Nat. Neurosci..

[56] Zhou Y., Ge Y., Liu Q., Li Y.X., Chao X., Guan J.J., Diwu Y.C., Zhang Q. (2021). LncRNA BACE1-AS Promotes Autophagy-Mediated Neuronal Damage Through The miR-214-3p/ATG5 Signalling Axis In Alzheimer’s Disease. Neuroscience.

[57] Ge Y., Song X., Liu J., Liu C., Xu C. (2020). The Combined Therapy of Berberine Treatment with lncRNA BACE1-AS Depletion Attenuates Aβ(25-35) Induced Neuronal Injury Through Regulating the Expression of miR-132-3p in Neuronal Cells. Neurochem. Res..

[58] Xue C., Li G., Lu J., Luo J., Jia J. (2021). Novel insights for lncRNA MAGI2-AS3 in solid tumors. Biomed. Pharmacother..

[59] Kai-Xin L., Cheng C., Rui L., Zheng-Wei S., Wen-Wen T., Peng X. (2021). Roles of lncRNA MAGI2-AS3 in human cancers. Biomed. Pharmacother..

[60] Cao C., Zhou S., Hu J. (2020). Long noncoding RNA MAGI2-AS3/miR-218-5p/GDPD5/SEC61A1 axis drives cellular proliferation and migration and confers cisplatin resistance in nasopharyngeal carcinoma. Int. Forum Allergy Rhinol..

[61] He J., Zhou X., Li L., Han Z. (2020). Long Noncoding MAGI2-AS3 Suppresses Several Cellular Processes of Lung Squamous Cell Carcinoma Cells by Regulating miR-374a/b-5p/CADM2 Axis. Cancer Manag. Res..

[62] Rastogi M., Singh S.K. (2019). Modulation of Type-I Interferon Response by hsa-miR-374b-5p During Japanese Encephalitis Virus Infection in Human Microglial Cells. Front. Cell. Infect. Microbiol..

[63] Booy E.P., McRae E.K., Howard R., Deo S.R., Ariyo E.O., Dzananovic E., Meier M., Stetefeld J., McKenna S.A. (2016). RNA Helicase Associated with AU-rich Element (RHAU/DHX36) Interacts with the 3’-Tail of the Long Non-coding RNA BC200 (BCYRN1). J. Biol. Chem..

[64] Tiedge H., Chen W., Brosius J. (1993). Primary structure, neural-specific expression, and dendritic location of human BC200 RNA. J. Neurosci. Off. J. Soc. Neurosci..

[65] Mus E., Hof P.R., Tiedge H. (2007). Dendritic BC200 RNA in aging and in Alzheimer’s disease. Proc. Natl. Acad. Sci. USA.

[66] Li H., Zheng L., Jiang A., Mo Y., Gong Q. (2018). Identification of the biological affection of long noncoding RNA BC200 in Alzheimer’s disease. Neuroreport.

[67] Zhang T., Pang P., Fang Z., Guo Y., Li H., Li X., Tian T., Yang X., Chen W., Shu S. (2018). Expression of BC1 Impairs Spatial Learning and Memory in Alzheimer’s Disease Via APP Translation. Mol. Neurobiol..

[68] Massone S., Vassallo I., Fiorino G., Castelnuovo M., Barbieri F., Borghi R., Tabaton M., Robello M., Gatta E., Russo C. (2011). 17A, a novel non-coding RNA, regulates GABA B alternative splicing and signaling in response to inflammatory stimuli and in Alzheimer disease. Neurobiol. Dis..

[69] Pickkers P., Mehta R.L., Murray P.T., Joannidis M., Molitoris B.A., Kellum J.A., Bachler M., Hoste E.A.J., Hoiting O., Krell K. (2018). Effect of Human Recombinant Alkaline Phosphatase on 7-Day Creatinine Clearance in Patients With Sepsis-Associated Acute Kidney Injury: A Randomized Clinical Trial. JAMA.

[70] Sun J., Zhao J., Bao X., Wang Q. (2018). Alkaline Phosphatase Assay Based on the Chromogenic Interaction of Diethanolamine with 4-Aminophenol. Anal. Chem..

[71] Heinrich D., Bruland Ø., Guise T.A., Suzuki H., Sartor O. (2018). Alkaline phosphatase in metastatic castration-resistant prostate cancer: Reassessment of an older biomarker. Future Oncol..

[72] Xie L., Zhang N., Zhang Q., Li C., Sandhu A.F., Iii G.W., Lin S., Lv P., Liu Y., Wu Q. (2020). Inflammatory factors and amyloid β-induced microglial polarization promote inflammatory crosstalk with astrocytes. Aging.

[73] Motoi Y., Aizawa T., Haga S., Nakamura S., Namba Y., Ikeda K. (1999). Neuronal localization of a novel mosaic apolipoprotein E receptor, LR11, in rat and human brain. Brain Res..

[74] Hermans-Borgmeyer I., Hampe W., Schinke B., Methner A., Nykjaer A., Süsens U., Fenger U., Herbarth B., Schaller H.C. (1998). Unique expression pattern of a novel mosaic receptor in the developing cerebral cortex. Mech. Dev..

[75] Jacobsen L., Madsen P., Nielsen M.S., Geraerts W.P., Gliemann J., Smit A.B., Petersen C.M. (2002). The sorLA cytoplasmic domain interacts with GGA1 and -2 and defines minimum requirements for GGA binding. FEBS Lett..

[76] Scherzer C.R., Offe K., Gearing M., Rees H.D., Fang G., Heilman C.J., Schaller C., Bujo H., Levey A.I., Lah J.J. (2004). Loss of apolipoprotein E receptor LR11 in Alzheimer disease. Arch. Neurol..

[77] Offe K., Dodson S.E., Shoemaker J.T., Fritz J.J., Gearing M., Levey A.I., Lah J.J. (2006). The lipoprotein receptor LR11 regulates amyloid beta production and amyloid precursor protein traffic in endosomal compartments. J. Neurosci. Off. J. Soc. Neurosci..

[78] Ma Q.L., Galasko D.R., Ringman J.M., Vinters H.V., Edland S.D., Pomakian J., Ubeda O.J., Rosario E.R., Teter B., Frautschy S.A. (2009). Reduction of SorLA/LR11, a sorting protein limiting beta-amyloid production, in Alzheimer disease cerebrospinal fluid. Arch. Neurol..

[79] Tsolakidou A., Alexopoulos P., Guo L.H., Grimmer T., Westerteicher C., Kratzer M., Jiang M., Bujo H., Roselli F., Leante M.R. (2013). β-Site amyloid precursor protein-cleaving enzyme 1 activity is related to cerebrospinal fluid concentrations of sortilin-related receptor with A-type repeats, soluble amyloid precursor protein, and tau. Alzheimer’s Dement. J. Alzheimer’s Assoc..

[80] Schmidt V., Baum K., Lao A., Rateitschak K., Schmitz Y., Teichmann A., Wiesner B., Petersen C.M., Nykjaer A., Wolf J. (2012). Quantitative modelling of amyloidogenic processing and its influence by SORLA in Alzheimer’s disease. EMBO J..

[81] Schmidt V., Sporbert A., Rohe M., Reimer T., Rehm A., Andersen O.M., Willnow T.E. (2007). SorLA/LR11 regulates processing of amyloid precursor protein via interaction with adaptors GGA and PACS-1. J. Biol. Chem..

[82] Caglayan S., Takagi-Niidome S., Liao F., Carlo A.S., Schmidt V., Burgert T., Kitago Y., Füchtbauer E.M., Füchtbauer A., Holtzman D.M. (2014). Lysosomal sorting of amyloid-β by the SORLA receptor is impaired by a familial Alzheimer’s disease mutation. Sci. Transl. Med..

[83] Gómez-Tortosa E., Ruggiero M., Sainz M.J., Villarejo-Galende A., Prieto-Jurczynska C., Venegas Pérez B., Ordás C., Agüero P., Guerrero-López R., Pérez-Pérez J. (2018). SORL1 Variants in Familial Alzheimer’s Disease. J. Alzheimer’s Dis. JAD.

[84] Nicolas G., Acuña-Hidalgo R., Keogh M.J., Quenez O., Steehouwer M., Lelieveld S., Rousseau S., Richard A.C., Oud M.S., Marguet F. (2018). Somatic variants in autosomal dominant genes are a rare cause of sporadic Alzheimer’s disease. Alzheimer’s Dement. J. Alzheimer’s Assoc..

[85] Ciarlo E., Massone S., Penna I., Nizzari M., Gigoni A., Dieci G., Russo C., Florio T., Cancedda R., Pagano A. (2013). An intronic ncRNA-dependent regulation of SORL1 expression affecting Aβ formation is upregulated in post-mortem Alzheimer’s disease brain samples. Dis. Model. Mech..

[86] Dieci G., Fiorino G., Castelnuovo M., Teichmann M., Pagano A. (2007). The expanding RNA polymerase III transcriptome. Trends Genet. TIG.

[87] Pagano A., Castelnuovo M., Tortelli F., Ferrari R., Dieci G., Cancedda R. (2007). New small nuclear RNA gene-like transcriptional units as sources of regulatory transcripts. PLoS Genet..

[88] De Preter K., Vandesompele J., Menten B., Carr P., Fiegler H., Edsjö A., Carter N.P., Yigit N., Waelput W., Van Roy N. (2005). Positional and functional mapping of a neuroblastoma differentiation gene on chromosome 11. BMC Genom..

[89] Amid C., Bahr A., Mujica A., Sampson N., Bikar S.E., Winterpacht A., Zabel B., Hankeln T., Schmidt E.R. (2001). Comparative genomic sequencing reveals a strikingly similar architecture of a conserved syntenic region on human chromosome 11p15.3 (including gene ST5) and mouse chromosome 7. Cytogenet. Cell Genet..

[90] Castelnuovo M., Massone S., Tasso R., Fiorino G., Gatti M., Robello M., Gatta E., Berger A., Strub K., Florio T. (2010). An Alu-like RNA promotes cell differentiation and reduces malignancy of human neuroblastoma cells. FASEB J. Off. Publ. Fed. Am. Soc. Exp. Biol..

[91] Gavazzo P., Vella S., Marchetti C., Nizzari M., Cancedda R., Pagano A. (2011). Acquisition of neuron-like electrophysiological properties in neuroblastoma cells by controlled expression of NDM29 ncRNA. J. Neurochem..

[92] Massone S., Ciarlo E., Vella S., Nizzari M., Florio T., Russo C., Cancedda R., Pagano A. (2012). NDM29, a RNA polymerase III-dependent non coding RNA, promotes amyloidogenic processing of APP and amyloid β secretion. Biochim. Biophys. Acta.

[93] Chao M.V. (2003). Neurotrophins and their receptors: A convergence point for many signalling pathways. Nat. Rev. Neurosci..

[94] Huang E.J., Reichardt L.F. (2001). Neurotrophins: Roles in neuronal development and function. Annu. Rev. Neurosci..

[95] Patapoutian A., Reichardt L.F. (2001). Trk receptors: Mediators of neurotrophin action. Curr. Opin. Neurobiol..

[96] Poo M.M. (2001). Neurotrophins as synaptic modulators. Nat. Rev. Neurosci..

[97] Tapia-Arancibia L., Aliaga E., Silhol M., Arancibia S. (2008). New insights into brain BDNF function in normal aging and Alzheimer disease. Brain Res. Rev..

[98] Autry A.E., Monteggia L.M. (2012). Brain-derived neurotrophic factor and neuropsychiatric disorders. Pharmacol. Rev..

[99] Connor B., Young D., Yan Q., Faull R.L., Synek B., Dragunow M. (1997). Brain-derived neurotrophic factor is reduced in Alzheimer’s disease. Brain Research. Mol. Brain Res..

[100] Ferrer I., Marín C., Rey M.J., Ribalta T., Goutan E., Blanco R., Tolosa E., Martí E. (1999). BDNF and full-length and truncated TrkB expression in Alzheimer disease. Implications in therapeutic strategies. J. Neuropathol. Exp. Neurol..

[101] Garzon D., Yu G., Fahnestock M. (2002). A new brain-derived neurotrophic factor transcript and decrease in brain-derived neurotrophic factor transcripts 1, 2 and 3 in Alzheimer’s disease parietal cortex. J. Neurochem..

[102] Hock C., Heese K., Hulette C., Rosenberg C., Otten U. (2000). Region-specific neurotrophin imbalances in Alzheimer disease: Decreased levels of brain-derived neurotrophic factor and increased levels of nerve growth factor in hippocampus and cortical areas. Arch. Neurol..

[103] Holsinger R.M., Schnarr J., Henry P., Castelo V.T., Fahnestock M. (2000). Quantitation of BDNF mRNA in human parietal cortex by competitive reverse transcription-polymerase chain reaction: Decreased levels in Alzheimer’s disease. Brain Res. Mol. Brain Res..

[104] Allen S.J., Watson J.J., Dawbarn D. (2011). The neurotrophins and their role in Alzheimer’s disease. Curr. Neuropharmacol..

[105] Phillips H.S., Hains J.M., Armanini M., Laramee G.R., Johnson S.A., Winslow J.W. (1991). BDNF mRNA is decreased in the hippocampus of individuals with Alzheimer’s disease. Neuron.

[106] Siegel G.J., Chauhan N.B. (2000). Neurotrophic factors in Alzheimer’s and Parkinson’s disease brain. Brain Res. Brain Res. Rev..

[107] Forlenza O.V., Diniz B.S., Gattaz W.F. (2010). Diagnosis and biomarkers of predementia in Alzheimer’s disease. BMC Med..

[108] Forlenza O.V., Diniz B.S., Talib L.L., Radanovic M., Yassuda M.S., Ojopi E.B., Gattaz W.F. (2010). Clinical and biological predictors of Alzheimer’s disease in patients with amnestic mild cognitive impairment. Rev. Bras. Psiquiatr..

[109] Forlenza O.V., Diniz B.S., Teixeira A.L., Ojopi E.B., Talib L.L., Mendonça V.A., Izzo G., Gattaz W.F. (2010). Effect of brain-derived neurotrophic factor Val66Met polymorphism and serum levels on the progression of mild cognitive impairment. World J. Biol. Psychiatry Off. J. World Fed. Soc. Biol. Psychiatry.

[110] Laske C., Stransky E., Leyhe T., Eschweiler G.W., Maetzler W., Wittorf A., Soekadar S., Richartz E., Koehler N., Bartels M. (2007). BDNF serum and CSF concentrations in Alzheimer’s disease, normal pressure hydrocephalus and healthy controls. J. Psychiatr. Res..

[111] Lee J.G., Shin B.S., You Y.S., Kim J.E., Yoon S.W., Jeon D.W., Baek J.H., Park S.W., Kim Y.H. (2009). Decreased serum brain-derived neurotrophic factor levels in elderly korean with dementia. Psychiatry Investig..

[112] Komulainen P., Pedersen M., Hänninen T., Bruunsgaard H., Lakka T.A., Kivipelto M., Hassinen M., Rauramaa T.H., Pedersen B.K., Rauramaa R. (2008). BDNF is a novel marker of cognitive function in ageing women: The DR’s EXTRA Study. Neurobiol. Learn. Mem..

[113] Laske C., Stransky E., Leyhe T., Eschweiler G.W., Wittorf A., Richartz E., Bartels M., Buchkremer G., Schott K. (2006). Stage-dependent BDNF serum concentrations in Alzheimer’s disease. J. Neural Transm..

[114] Michalski B., Fahnestock M. (2003). Pro-brain-derived neurotrophic factor is decreased in parietal cortex in Alzheimer’s disease. Brain Res. Mol. Brain Res..

[115] Garzon D.J., Fahnestock M. (2007). Oligomeric amyloid decreases basal levels of brain-derived neurotrophic factor (BDNF) mRNA via specific downregulation of BDNF transcripts IV and V in differentiated human neuroblastoma cells. J. Neurosci. Off. J. Soc. Neurosci..

[116] Christensen R., Marcussen A.B., Wörtwein G., Knudsen G.M., Aznar S. (2008). Abeta(1-42) injection causes memory impairment, lowered cortical and serum BDNF levels, and decreased hippocampal 5-HT(2A) levels. Exp. Neurol..

[117] Ciaramella A., Salani F., Bizzoni F., Orfei M.D., Langella R., Angelucci F., Spalletta G., Taddei A.R., Caltagirone C., Bossù P. (2013). The stimulation of dendritic cells by amyloid beta 1-42 reduces BDNF production in Alzheimer’s disease patients. Brain Behav. Immun..

[118] Arancibia S., Silhol M., Moulière F., Meffre J., Höllinger I., Maurice T., Tapia-Arancibia L. (2008). Protective effect of BDNF against beta-amyloid induced neurotoxicity in vitro and in vivo in rats. Neurobiol. Dis..

[119] Liu Q.R., Walther D., Drgon T., Polesskaya O., Lesnick T.G., Strain K.J., de Andrade M., Bower J.H., Maraganore D.M., Uhl G.R. (2005). Human brain derived neurotrophic factor (BDNF) genes, splicing patterns, and assessments of associations with substance abuse and Parkinson’s Disease. Am. J. Med. Genetics. Part B Neuropsychiatr. Genet. Off. Publ. Int. Soc. Psychiatr. Genet..

[120] Modarresi F., Faghihi M.A., Lopez-Toledano M.A., Fatemi R.P., Magistri M., Brothers S.P., van der Brug M.P., Wahlestedt C. (2012). Inhibition of natural antisense transcripts in vivo results in gene-specific transcriptional upregulation. Nat. Biotechnol..

[121] Ding Y., Luan W., Shen X., Wang Z., Cao Y. (2022). LncRNA BDNF-AS as ceRNA regulates the miR-9-5p/BACE1 pathway affecting neurotoxicity in Alzheimer’s disease. Arch. Gerontol. Geriatr..

[122] Guo C.C., Jiao C.H., Gao Z.M. (2018). Silencing of LncRNA BDNF-AS attenuates Aβ(25-35)-induced neurotoxicity in PC12 cells by suppressing cell apoptosis and oxidative stress. Neurol. Res..

[123] Van Gool B., Storck S.E., Reekmans S.M., Lechat B., Gordts P., Pradier L., Pietrzik C.U., Roebroek A.J.M. (2019). LRP1 Has a Predominant Role in Production over Clearance of Aβ in a Mouse Model of Alzheimer’s Disease. Mol. Neurobiol..

[124] Kanekiyo T., Cirrito J.R., Liu C.C., Shinohara M., Li J., Schuler D.R., Shinohara M., Holtzman D.M., Bu G. (2013). Neuronal clearance of amyloid-β by endocytic receptor LRP1. J. Neurosci. Off. J. Soc. Neurosci..

[125] Liu C.C., Hu J., Zhao N., Wang J. (2017). Astrocytic LRP1 Mediates Brain Aβ Clearance and Impacts Amyloid Deposition. J. Neurosci. Res..

[126] Urmoneit B., Prikulis I., Wihl G., D’Urso D., Frank R., Heeren J., Beisiegel U., Prior R. (1997). Cerebrovascular smooth muscle cells internalize Alzheimer amyloid beta protein via a lipoprotein pathway: Implications for cerebral amyloid angiopathy. Lab. Investig. J. Tech. Methods Pathol..

[127] Mandrekar S., Jiang Q., Lee C.Y., Koenigsknecht-Talboo J., Holtzman D.M., Landreth G.E. (2009). Microglia mediate the clearance of soluble Abeta through fluid phase macropinocytosis. J. Neurosci. Off. J. Soc. Neurosci..

[128] Storck S.E., Hartz A.M.S., Bernard J., Wolf A., Kachlmeier A., Mahringer A., Weggen S., Pahnke J., Pietrzik C.U. (2018). The concerted amyloid-beta clearance of LRP1 and ABCB1/P-gp across the blood-brain barrier is linked by PICALM. Brain Behav. Immun..

[129] Yoon S.S., Jo S.A. (2012). Mechanisms of Amyloid-β Peptide Clearance: Potential Therapeutic Targets for Alzheimer’s Disease. Biomol. Ther..

[130] von Einem B., Schwanzar D., Rehn F., Beyer A.S., Weber P., Wagner M., Schneckenburger H., von Arnim C.A. (2010). The role of low-density receptor-related protein 1 (LRP1) as a competitive substrate of the amyloid precursor protein (APP) for BACE1. Exp. Neurol..

[131] Rebeck G.W., Reiter J.S., Strickland D.K., Hyman B.T. (1993). Apolipoprotein E in sporadic Alzheimer’s disease: Allelic variation and receptor interactions. Neuron.

[132] Yamanaka Y., Faghihi M.A., Magistri M., Alvarez-Garcia O., Lotz M., Wahlestedt C. (2015). Antisense RNA controls LRP1 Sense transcript expression through interaction with a chromatin-associated protein, HMGB2. Cell Rep..

[133] West J.A., Davis C.P., Sunwoo H., Simon M.D., Sadreyev R.I., Wang P.I., Tolstorukov M.Y., Kingston R.E. (2014). The long noncoding RNAs NEAT1 and MALAT1 bind active chromatin sites. Mol. Cell.

[134] Spreafico M., Grillo B., Rusconi F., Battaglioli E., Venturin M. (2018). Multiple Layers of CDK5R1 Regulation in Alzheimer’s Disease Implicate Long Non-Coding RNAs. Int. J. Mol. Sci..

[135] Ke S., Yang Z. (2019). Long Noncoding RNA NEAT1 Aggravates Aβ-Induced Neuronal Damage by Targeting miR-107 in Alzheimer’s Disease. Yonsei Med. J..

[136] Zhao M.Y., Wang G.Q., Wang N.N., Yu Q.Y., Liu R.L., Shi W.Q. (2019). The long-non-coding RNA NEAT1 is a novel target for Alzheimer’s disease progression via miR-124/BACE1 axis. Neurol. Res..

[137] Wang Z., Zhao Y., Xu N., Zhang S., Wang S., Mao Y., Zhu Y., Li B., Jiang Y., Tan Y. (2019). NEAT1 regulates neuroglial cell mediating Aβ clearance via the epigenetic regulation of endocytosis-related genes expression. Cell. Mol. Life Sci. CMLS.

[138] Raposo A.C., Casanova M., Gendrel A.V. (2021). The tandem repeat modules of Xist lncRNA: A swiss army knife for the control of X-chromosome inactivation. Biochem. Soc. Trans..

[139] Wang W., Min L., Qiu X., Wu X., Liu C., Ma J., Zhang D., Zhu L. (2021). Biological Function of Long Non-coding RNA (LncRNA) Xist. Front. Cell Dev. Biol..

[140] Chanda K., Mukhopadhyay D. (2020). LncRNA Xist, X-chromosome Instability and Alzheimer’s Disease. Curr. Alzheimer Res..

[141] Krittanawong C., Kitai T. (2017). Pharmacogenomics of angiotensin receptor/neprilysin inhibitor and its long-term side effects. Cardiovasc. Ther..

[142] Grimm M.O., Mett J., Stahlmann C.P., Haupenthal V.J., Zimmer V.C., Hartmann T. (2013). Neprilysin and Aβ Clearance: Impact of the APP Intracellular Domain in NEP Regulation and Implications in Alzheimer’s Disease. Front. Aging Neurosci..

[143] Nalivaeva N.N., Zhuravin I.A., Turner A.J. (2020). Neprilysin expression and functions in development, ageing and disease. Mech. Ageing Dev..

[144] Yan X.W., Liu H.J., Hong Y.X., Meng T., Du J., Chang C. (2022). LncRNA XIST induces Aβ accumulation and neuroinflammation by the epigenetic repression of NEP in Alzheimer’s disease. J. Neurogenet..

[145] Yue D., Guanqun G., Jingxin L., Sen S., Shuang L., Yan S., Minxue Z., Ping Y., Chong L., Zhuobo Z. (2020). Silencing of long noncoding RNA XIST attenuated Alzheimer’s disease-related BACE1 alteration through miR-124. Cell Biol. Int..

[146] Li J., Li Z., Zheng W., Li X., Wang Z., Cui Y., Jiang X. (2017). LncRNA-ATB: An indispensable cancer-related long noncoding RNA. Cell Prolif..

[147] Wang J., Zhou T., Wang T., Wang B. (2018). Suppression of lncRNA-ATB prevents amyloid-β-induced neurotoxicity in PC12 cells via regulating miR-200/ZNF217 axis. Biomed. Pharmacother..

[148] Li Y., Wu H., Wang Q., Xu S. (2021). ZNF217: The cerberus who fails to guard the gateway to lethal malignancy. Am. J. Cancer Res..

[149] Okazaki Y., Furuno M., Kasukawa T., Adachi J., Bono H., Kondo S., Nikaido I., Osato N., Saito R., Suzuki H. (2002). Analysis of the mouse transcriptome based on functional annotation of 60,770 full-length cDNAs. Nature.

[150] Cai Y., Sun Z., Jia H., Luo H., Ye X., Wu Q., Xiong Y., Zhang W., Wan J. (2017). Rpph1 Upregulates CDC42 Expression and Promotes Hippocampal Neuron Dendritic Spine Formation by Competing with miR-330-5p. Front. Mol. Neurosci..

[151] Gu R., Liu R., Wang L., Tang M., Li S.R., Hu X. (2021). LncRNA RPPH1 attenuates Aβ(25-35)-induced endoplasmic reticulum stress and apoptosis in SH-SY5Y cells via miR-326/PKM2. Int. J. Neurosci..

[152] Gu R., Wang L., Tang M., Li S.R., Liu R., Hu X. (2020). LncRNA Rpph1 protects amyloid-β induced neuronal injury in SK-N-SH cells via miR-122/Wnt1 axis. Int. J. Neurosci..

[153] Liu L., Wan W., Xia S., Kalionis B., Li Y. (2014). Dysfunctional Wnt/β-catenin signaling contributes to blood-brain barrier breakdown in Alzheimer’s disease. Neurochem. Int..

[154] Yi R., Chen B., Zhao J., Zhan X., Zhang L., Liu X., Dong Q. (2014). Krüppel-like factor 8 ameliorates Alzheimer’s disease by activating β-catenin. J. Mol. Neurosci. MN.

[155] Ratajczak M.Z. (2012). Igf2-H19, an imprinted tandem gene, is an important regulator of embryonic development, a guardian of proliferation of adult pluripotent stem cells, a regulator of longevity, and a ‘passkey’ to cancerogenesis. Folia Histochem. Cytobiol..

[156] Gabory A., Ripoche M.A., Yoshimizu T., Dandolo L. (2006). The H19 gene: Regulation and function of a non-coding RNA. Cytogenet. Genome Res..

[157] Jia Y.M., Zhu C.F., Yang K., He C.G., Wu Y.Y., Wang L., Song R.F., Zhang J.Y., Wang C. (2022). Effect of moxibustion on autophagy lysosome function mediated by mTOR/TFEB pathway and lncRNA H19 expression in APP/PS1 double transgenic mice. Zhen Ci Yan Jiu Acupunct. Res..

[158] Zhang Y.Y., Bao H.L., Dong L.X., Liu Y., Zhang G.W., An F.M. (2021). Silenced lncRNA H19 and up-regulated microRNA-129 accelerates viability and restrains apoptosis of PC12 cells induced by Aβ(25-35) in a cellular model of Alzheimer’s disease. Cell Cycle.

[159] Zong S., Dai W., Guo X., Wang K. (2021). LncRNA-SNHG1 promotes macrophage M2-like polarization and contributes to breast cancer growth and metastasis. Aging.

[160] Gao Y., Zhang N., Lv C., Li N., Li X., Li W. (2020). LncRNA SNHG1 Knockdown Alleviates Amyloid-β-Induced Neuronal Injury by Regulating ZNF217 via Sponging miR-361-3p in Alzheimer’s Disease. J. Alzheimer’s Dis. JAD.

[161] Wang H., Lu B., Chen J. (2019). Knockdown of lncRNA SNHG1 attenuated Aβ(25-35)-inudced neuronal injury via regulating KREMEN1 by acting as a ceRNA of miR-137 in neuronal cells. Biochem. Biophys. Res. Commun..

[162] Moorwood K., Charles A.K., Salpekar A., Wallace J.I., Brown K.W., Malik K. (1998). Antisense WT1 transcription parallels sense mRNA and protein expression in fetal kidney and can elevate protein levels in vitro. J. Pathol..

[163] Malik K., Salpekar A., Hancock A., Moorwood K., Jackson S., Charles A., Brown K.W. (2000). Identification of differential methylation of the WT1 antisense regulatory region and relaxation of imprinting in Wilms’ tumor. Cancer Res..

[164] Wang Q., Ge X., Zhang J., Chen L. (2020). Effect of lncRNA WT1-AS regulating WT1 on oxidative stress injury and apoptosis of neurons in Alzheimer’s disease via inhibition of the miR-375/SIX4 axis. Aging.

[165] Magistri M., Velmeshev D., Makhmutova M., Faghihi M.A. (2015). Transcriptomics Profiling of Alzheimer’s Disease Reveal Neurovascular Defects, Altered Amyloid-β Homeostasis, and Deregulated Expression of Long Noncoding RNAs. J. Alzheimer’s Dis. JAD.

[166] Gu C., Chen C., Wu R., Dong T., Hu X., Yao Y., Zhang Y. (2018). Long Noncoding RNA EBF3-AS Promotes Neuron Apoptosis in Alzheimer’s Disease. DNA Cell Biol..

[167] Xiao Y., Li Y., Zhang H., Yang L., Jiang Y., Wei C., Feng X., Xun Y., Yuan S., Xiang S. (2021). TNFAIP1 Is Upregulated in APP/PS1 Mice and Promotes Apoptosis in SH-SY5Y Cells by Binding to RhoB. J. Mol. Neurosci. MN.

[168] Qiu F., Zhou Y., Deng Y., Yi J., Gong M., Liu N., Wei C., Xiang S. (2020). Knockdown of TNFAIP1 prevents di-(2-ethylhexyl) phthalate-induced neurotoxicity by activating CREB pathway. Chemosphere.

[169] Yi J., Zhu M., Qiu F., Zhou Y., Shu P., Liu N., Wei C. (2020). TNFAIP1 Mediates Formaldehyde-Induced Neurotoxicity by Inhibiting the Akt/CREB Pathway in N2a Cells. Neurotox. Res..

[170] Li Y., Jin L., Wang F., Ren L., Pen R., Bo G., Wang L. (2022). Epigenetic axis of SNHG19/miR-137/TNFAIP1 modulates amyloid beta peptide 25-35-induced SH-SY5Y cytotoxicity. Epigenomics.

[171] Yang C.M., Wang T.H., Chen H.C., Li S.C., Lee M.C., Liou H.H., Liu P.F., Tseng Y.K., Shiue Y.L., Ger L.P. (2016). Aberrant DNA hypermethylation-silenced SOX21-AS1 gene expression and its clinical importance in oral cancer. Clin. Epigenet..

[172] Zhang L., Fang Y., Cheng X., Lian Y.J., Xu H.L. (2019). Silencing of Long Noncoding RNA SOX21-AS1 Relieves Neuronal Oxidative Stress Injury in Mice with Alzheimer’s Disease by Upregulating FZD3/5 via the Wnt Signaling Pathway. Mol. Neurobiol..

[173] Xu W., Li K., Fan Q., Zong B., Han L. (2020). Knockdown of long non-coding RNA SOX21-AS1 attenuates amyloid-β-induced neuronal damage by sponging miR-107. Biosci. Rep..

[174] Gu F., Ji D., Ni H., Chen D. (2021). SRY-Box 21 Antisense RNA 1 Knockdown Diminishes Amyloid Beta(25-35)-Induced Neuronal Damage by miR-132/PI3K/AKT Pathway. Neurochem. Res..

[175] Zhou Y., Tian B., Tang J., Wu J., Wang H., Wu Z., Li X., Yang D., Zhang B., Xiao Y. (2020). SNHG7: A novel vital oncogenic lncRNA in human cancers. Biomed. Pharmacother..

[176] Freeze W.M., Bacskai B.J., Frosch M.P., Jacobs H.I.L., Backes W.H., Greenberg S.M., van Veluw S.J. (2019). Blood-Brain Barrier Leakage and Microvascular Lesions in Cerebral Amyloid Angiopathy. Stroke.

[177] Biron K.E., Dickstein D.L., Gopaul R., Jefferies W.A. (2011). Amyloid triggers extensive cerebral angiogenesis causing blood brain barrier permeability and hypervascularity in Alzheimer’s disease. PLoS ONE.

[178] Li J.R., Zhao Y.S., Chang Y., Yang S.C., Guo Y.J., Ji E.S. (2018). Fasudil improves endothelial dysfunction in rats exposed to chronic intermittent hypoxia through RhoA/ROCK/NFATc3 pathway. PLoS ONE.

[179] Garcia-Vaz E., McNeilly A.D., Berglund L.M., Ahmad A., Gallagher J.R., Dutius Andersson A.M., McCrimmon R.J. (2020). Inhibition of NFAT Signaling Restores Microvascular Endothelial Function in Diabetic Mice. Diabetes.

[180] Ning H., Zhang L., Zhu B., Zhou X., Zhang T., Ma T. (2022). TARBP2-stablized SNHG7 regulates blood-brain barrier permeability by acting as a competing endogenous RNA to miR-17-5p/NFATC3 in Aβ-microenvironment. Cell Death Dis..

[181] Razeghian-Jahromi I., Karimi Akhormeh A. (2022). The Role of ANRIL in Atherosclerosis. Dis. Markers.

[182] Zhou B., Li L., Qiu X., Wu J., Xu L., Shao W. (2020). Long non-coding RNA ANRIL knockdown suppresses apoptosis and pro-inflammatory cytokines while enhancing neurite outgrowth via binding microRNA-125a in a cellular model of Alzheimer’s disease. Mol. Med. Rep..

[183] Ji P., Diederichs S., Wang W., Böing S., Metzger R., Schneider P.M., Tidow N., Brandt B., Buerger H., Bulk E. (2003). MALAT-1, a novel noncoding RNA, and thymosin beta4 predict metastasis and survival in early-stage non-small cell lung cancer. Oncogene.

[184] Jin Y., Feng S.J., Qiu S., Shao N., Zheng J.H. (2017). LncRNA MALAT1 promotes proliferation and metastasis in epithelial ovarian cancer via the PI3K-AKT pathway. Eur. Rev. Med. Pharmacol. Sci..

[185] Zhang B., Arun G., Mao Y.S., Lazar Z., Hung G., Bhattacharjee G., Xiao X., Booth C.J., Wu J., Zhang C. (2012). The lncRNA Malat1 is dispensable for mouse development but its transcription plays a cis-regulatory role in the adult. Cell Rep..

[186] Bernard D., Prasanth K.V., Tripathi V., Colasse S., Nakamura T., Xuan Z., Zhang M.Q., Sedel F., Jourdren L., Coulpier F. (2010). A long nuclear-retained non-coding RNA regulates synaptogenesis by modulating gene expression. EMBO J..

[187] Masoumi F., Ghorbani S., Talebi F., Branton W.G., Rajaei S., Power C., Noorbakhsh F. (2019). Malat1 long noncoding RNA regulates inflammation and leukocyte differentiation in experimental autoimmune encephalomyelitis. J. Neuroimmunol..

[188] Yao J., Wang X.Q., Li Y.J., Shan K., Yang H., Wang Y.N., Yao M.D., Liu C., Li X.M., Shen Y. (2016). Long non-coding RNA MALAT1 regulates retinal neurodegeneration through CREB signaling. EMBO Mol. Med..

[189] Ma P., Li Y., Zhang W., Fang F., Sun J., Liu M., Li K., Dong L. (2019). Long Non-coding RNA MALAT1 Inhibits Neuron Apoptosis and Neuroinflammation While Stimulates Neurite Outgrowth and Its Correlation With MiR-125b Mediates PTGS2, CDK5 and FOXQ1 in Alzheimer’s Disease. Curr. Alzheimer Res..

[190] Li L., Xu Y., Zhao M., Gao Z. (2020). Neuro-protective roles of long non-coding RNA MALAT1 in Alzheimer’s disease with the involvement of the microRNA-30b/CNR1 network and the following PI3K/AKT activation. Exp. Mol. Pathol..

[191] Shafi O. (2016). Inverse relationship between Alzheimer’s disease and cancer, and other factors contributing to Alzheimer’s disease: A systematic review. BMC Neurol..

[192] Chanda K., Jana N.R., Mukhopadhyay D. (2022). Long non-coding RNA MALAT1 protects against Aβ(1-42) induced toxicity by regulating the expression of receptor tyrosine kinase EPHA2 via quenching miR-200a/26a/26b in Alzheimer’s disease. Life Sci..

[193] Iwakiri J., Terai G., Hamada M. (2017). Computational prediction of lncRNA-mRNA interactionsby integrating tissue specificity in human transcriptome. Biol. Direct.

[194] Bennett C.F., Krainer A.R., Cleveland D.W. (2019). Antisense Oligonucleotide Therapies for Neurodegenerative Diseases. Annu. Rev. Neurosci..

[195] Modarresi F., Faghihi M.A., Patel N.S., Sahagan B.G., Wahlestedt C., Lopez-Toledano M.A. (2011). Knockdown of BACE1-AS Nonprotein-Coding Transcript Modulates Beta-Amyloid-Related Hippocampal Neurogenesis. Int. J. Alzheimer’s Dis..

[196] Barucker C., Harmeier A., Weiske J., Fauler B., Albring K.F., Prokop S., Hildebrand P., Lurz R., Heppner F.L., Huber O. (2014). Nuclear translocation uncovers the amyloid peptide Aβ42 as a regulator of gene transcription. J. Biol. Chem..

[197] Chanda K., Jana N.R., Mukhopadhyay D. (2021). Receptor tyrosine kinase ROR1 ameliorates Aβ(1-42) induced cytoskeletal instability and is regulated by the miR146a-NEAT1 nexus in Alzheimer’s disease. Sci. Rep..

[198] Wang X., Wang C., Geng C., Zhao K. (2018). LncRNA XIST knockdown attenuates Aβ(25-35)-induced toxicity, oxidative stress, and apoptosis in primary cultured rat hippocampal neurons by targeting miR-132. Int. J. Clin. Exp. Pathol..

